# Cost-Effectiveness of Rural Incentive Packages for Graduating Medical Students in Lao PDR

**DOI:** 10.15171/ijhpm.2016.141

**Published:** 2016-10-29

**Authors:** Eric Keuffel, Wanda Jaskiewicz, Khampasong Theppanya, Kate Tulenko

**Affiliations:** ^1^Health Finance & Access Initiative, Bryn Mawr, PA, USA.; ^2^IntraHealth International, Washington, DC, USA.; ^3^Ministry of Health (Lao PDR), Vientiane, Lao PDR.

**Keywords:** Health Workers (Rural), Health Economics (Cost-Effectiveness Analysis), Discrete Choice Experiment (DCE)

## Abstract

**Background:** The dearth of health workers in rural settings in Lao People’s Democratic Republic (PDR) and other developing countries limits healthcare access and outcomes. In evaluating non-wage financial incentive packages as a potential policy option to attract health workers to rural settings, understanding the expected costs and effects of the various programs ex ante can assist policy-makers in selecting the optimal incentive package.

**Methods:** We use discrete choice experiments (DCEs), costing analyses and recent empirical results linking health worker density and health outcomes to estimate the future location decisions of physicians and determine the cost-effectiveness of 15 voluntary incentives packages for new physicians in Lao PDR. Our data sources include a DCE survey completed by medical students (n = 329) in May 2011 and secondary cost, economic and health data. Mixed logit regressions provide the basis for estimating how each incentive package influences rural versus urban location choice over time. We estimate the expected rural density of physicians and the cost-effectiveness of 15 separate incentive packages from a societal perspective. In order to generate the cost-effectiveness ratios we relied on the rural uptake probabilities inferred from the DCEs, the costing data and prior World Health Organization (WHO) estimates that relate health outcomes to health worker density.

**Results**: Relative to no program, the optimal voluntary incentive package would increase rural physician density by 15% by 2016 and 65% by 2041. After incorporating anticipated health effects, seven (three) of the 15 incentive packages have anticipated average cost-effectiveness ratio less than the WHO threshold (three times gross domestic product [GDP] per capita) over a 5-year (30 year) period. The optimal package’s incremental cost-effectiveness ratio is $1454/QALY (quality-adjusted life year) over 5 years and $2380/QALY over 30 years. Capital intensive components, such as housing or facility improvement, are not efficient.

**Conclusion:** Conditional on using voluntary incentives, Lao PDR should emphasize non-capital intensive options such as advanced career promotion, transport subsidies and housing allowances to improve physician distribution and rural health outcomes in a cost-effective manner. Other countries considering voluntary incentive programs can implement health worker/trainee DCEs and costing surveys to determine which incentive bundles improve rural uptake most efficiently but should be aware of methodological caveats.

## Background

### Health Worker Production, Distribution and Population Health

Policy-makers, ministries of health (MoHs), donors, multilateral institutions and healthcare non-governmental organizations (NGOs) have increasingly recognized the importance of health workers as a critical input for improvement of population health outcomes over the last decade.^1-5^ In developing countries the limitations in production, productivity and output of health workers, or human resources for health (HRH), occurs as a result of several factors. First, the overall number of health workers, particularly in parts of Africa and Asia, is insufficient, or even critically low, according to public health, needs-based estimates and economic demand-based calculations.^6-8^ Second, the appropriate shares of health worker cadres expected to optimize health outcomes within the budget constraints of developing countries is poorly understood – particularly as technology developments are shifting the relative capabilities of different types of health workers. Finally, the health workforce, particularly for physicians, is geographically skewed. Many health workers from developing countries emigrate to other nations,^9^ but, just as in the developed world, remaining health workers are located in urban areas at rates greater than the urban population share.^10^ Within developing countries, the density of health workers relative to the population is typically significantly lower in rural areas than in urban areas.

In order to attract new or existing health workers into rural areas, policy-makers have considered (and in some cases adopted) both wage-based incentives (eg, higher salaries) and non-wage benefits (housing, accelerated promotion in the public staffing system, education stipends for children, access to better facilities and other non-salary inducements) or combinations of both.^11-15^ For countries examining implementation of rural incentive strategies, mapping out the expected affect that the incentive packages may have on location choice is central. For example, recent discrete choice experiments (DCE) supported by Capacity*Plus,* the US Agency for International Development (USAID)-funded global HRH project, among health workers and students training to become health workers indicate that both wage and non-wage determinants significantly impact location decision.^16-18^

DCEs, which can yield measures of utility, uptake probability or value; are increasingly used in health services research and health economics applications including studies aiming to estimate: (*a*) willingness to pay, (*b*) utility weights for quality-adjusted life year (QALY) measurement, and (*c*) probabilities and relative risk.^19-21^ These are applied across a variety of policy settings including (*a*) health professional choices (*b*) patient or clinician clinical choices, and (*c*) priority setting frameworks and are increasingly popular in developing country settings.^18,22-26^ Within health services research, DCEs have frequently been implemented to understand health workforce issues including applications aimed at improving the distribution of health workers in developing country settings.^25,27-34^ More recently novel studies have combined the uptake probabilities from health worker DCEs and secondary costing data in dynamic simulations to evaluate both the health worker location outcomes and associated costs of different policy options.^33,34^

This study uniquely contributes to the DCE literature by combining the results of a DCE study with a costing analysis and health outcome projections. First, we use the mixed logit estimates from the DCE to determine the implied value (or willingness to pay) for each component of rural incentive packages intended to increase the attractiveness of practicing in rural locations. Second, we compare the implied valuations from the DCE with the actual expected cost to the payer (in this case the Lao PDR Government) for each of the components as reported in a separate costing exercise. Lastly, we use uptake probabilities derived from the DCE to project the degree to which various proposed incentive packages would likely motivate additional rural entry by new physicians over a 5 (and 30) year period and combine these estimates with emerging literature which calculates the effects of health worker density on health outcomes to generate both average and incremental cost-effectiveness ratios of 15 different voluntary rural incentive packages proposed for Lao PDR.

### The Lao People’s Democratic Republic Human Resources for Health Context

Lao PDR, the setting for our study, is a lower-middle income (2011 gross domestic product [GDP] per capital, $1281) landlocked county with a population of 6.4 million (2011), primarily rural, citizens.^35^ As is the case with many lower-middle income countries, health expenditure is a relatively low share of GDP (4.1% in 2011), out-of-pocket expenditure are high (63% of all health expenditure, 2011) and density of health workers is low (2.4 physicians and 7.5 nurses per 10 000 population, 2011).^36^ The Lao Government, MoH, and multilateral partners have prioritized improvement in access to primary care with particular emphasis on maternal and child health. To this end, several recent initiatives have focused on increasing the number and distribution of health workers, particularly in rural settings where access to care is more challenging.^36^ In 2010, the Lao MoH adopted the Health Personnel Development Strategy (HPDS) for 2020 which set the framework and strategy which aimed to ensure that sufficient quality and quantity of health worker personnel are placed in appropriate in leadership, managerial and technical levels in the right geographic distribution with enough support and motivation to optimally perform their work.^16^ One of the central tenets of the HPDS was to ensure appropriate incentives for health workers – a task which the MoH Department of Organization and Personnel (DOP) sought to define further with development of a national HRH retention strategy inclusive of specific incentives and interventions aimed to increase work in rural areas.^16^ The survey for our study was an important component of determining which incentives would motivate health workers most significantly.

Two-thirds of the Lao population resides in rural areas, but less than one quarter of public sector physicians practice in rural settings (Table 1).^37,38^ We focus on physicians in our study given that the differential between the population and health worker concentration is largest for physicians in Lao PDR. The lower density of health workers reduces access to health services in these areas and evidence suggests the skewed distribution of physicians and health workers harms health outcomes.^39^

**Table 1:** Distribution of Health Workers in the Lao PDR Public System (2011)

	**MDs**	**MAs**	**Nurses/Midwives**	**Population (1000s)**
Number	1187	1463	5552	6275
Number Rural	273	693	3324	4131
Rural Share	0.23	0.47	0.60	0.66

Sources: WHO, Laos Country Profile 2012; UN Department of Economic and Social Affairs, World Urbanization Prospects 2011 (Accessed July 2013). Abbreviations: MA, medical assistant; PDR, People’s Democratic Republic; MD, doctor of Medicine.

The public sector is particularly important with respect to rural access since (*a*) public sector physicians comprise about 70% of total physicians and (*b*) few private sector facilities are located in urban areas.^40^ With respect to production of new physicians, Lao PDR has one 6-year medical school, the University of Health Sciences Faculty of Medical Sciences, based in the capital city, Vientiane. There are limited number of new physicians generated each year. The survey target initially was aimed to reach all of the approximately 360 fifth and sixth year students – so about 180 graduates (minus attrition) are anticipated to join the health system per year.^16^

The policy options for Lao PDR to improve distribution of health workers include (*a*) voluntary programs that use non-wage incentives such as housing, transportation or other perks to motivate health workers to locate in rural areas, (*b*) salary increases, (*c*) mandatory service, and (*d*) combinations of these choices for both practicing physicians, recent graduates or both sets of workers. This analytical work focuses on the evaluation of voluntary non-wage incentives for recent graduates and was conducted prior to the decision by the Lao PDR MoH to implement a mandatory 3-year placement of new physicians into rural settings. Specifically, our objective was to identify the most cost-effective of 15 non-wage voluntary rural incentive packages for new physicians proposed for Lao PDR. While we focused on Lao PDR context, the methods, limitations, and results are informative for many other countries considering how to optimally anticipate and understand how their health workers and trainees will decide to locate under various policies.

## Methods

### Discrete Choice Experiment Instrument Development and Administration

The attributes and levels for the DCE were developed via a review of literature and qualitative focus group discussions with medical students and interviews with key stakeholders from the Lao PDR MoH.^16,41^ On the basis of the responses the final version included six attributes: salary (4 levels: 50% increase, 40% increase, 30% increase, no increase), housing (3 levels: provision of house, allowance, no subsidy), transportation (3 levels: motorbike for work/leisure, motorbike for work only, no transportation benefit), career promotion (3 levels: immediate promotion to permanent staff upon posting, promotion after one year, promotion after two years), continuing education (3 levels: qualify for scholarship after one year, after two years, after three years) and facility quality (2 levels: improved vs. not improved). The limited subset of DCE scenario alternatives across these attribute levels was generated via use of Sawtooth Software’s Choice-Based Conjoint package (Sawtooth Software Inc., 2007). This software maximizes D-efficiency optimization to ensure level balance (each attribute is shown an equal number of times) and orthogonality (attribute levels are independent of other attribute levels). The final survey instrument offered a series of 12 questions in which respondents chose between two hypothetical job postings with varying attribute levels. Additionally, the instrument obtained information on demographics, education, and background (Technical Appendix: Exhibits 1 and 2 for Survey and Design Efficiency Report).^16^ Based on the DCE attributes, we focused on 15 incentive packages (Table 2).

**Table 2:** Incentive Package Components Based on Lao PDR DCE (Medical Students, n = 329)

**Incentive Package**	**Components in Select Packages (Future Physicians)**					
	**Housing (Construction)**	**Housing (Allowance)**	**Transport**	**Career Promotion**	**Additional Training**	**Facility Improvement**
1	•		•^OP^	•	•^1^	
2	•		•^O^	•	•^1^	
3	•		•^O^		•^1^	
4		•	•^OP^	•		
5			•^OP^		•^1^	•
6	•		•^O^	•		
7			•^OP^		•^1^	
8			•^O^		•^1^	
9			•^O^	•		•
10			•^OP^		•^2^	
11			•^O^	•		
12			•^O^		•^2^	
13		•			•^2^	
14	•				•^2^	
15					•^1^	

Abbreviations: DCE, discrete choice experiment; PDR, People’s Democratic Republic. •^OP^ Transport provided for official and personal use; ^O^ Transport for official use only; •^1^ Additional training available after one year of service; •^2^ Additional training available after two years of service.

### Administrations of Discrete Choice Experiment

**Figure 1:**
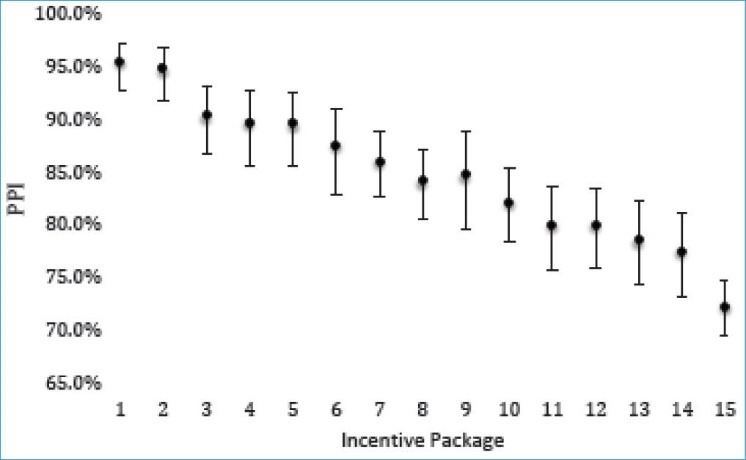
Predicted Preference Impact (PPI) Estimates and 95% CIs for each Incentive Package. PPI represents the probability of accepting the incentive package (relative to salary alone):

The data collection team included 15 members from the Lao PDR MoH and the World Health Organization (WHO) that subdivided into three smaller teams, one per province. Within each province the teams divided into two smaller teams for data collection activities. Prior to collection, teams participated in a one-day training in the application of the DCE survey and data collection protocols. All DCE surveys were administered to groups of respondents within a facility meeting room, classroom, or lecture hall using paper-based questionnaires. Each individual completed their survey independently and students indicated preferences for attributes related to 15 different incentive packages (Figure 1). The average survey completion time was approximately 20-30 minutes. Data collection occurred over a period of five days in May 2011. All respondents provided consent prior to participating in the study. Data was entered into Microsoft Excel and cross checked twice. The Lao PDR MoH gave ethical approval for the collection and use of data as part of efforts for health reform. Data were de-identified prior to analysis.

### Discrete Choice Experiment Medical Student Sample

As is the case in many international medical schools, medical students in Lao PDR enroll in a 6-year program combining undergraduate and graduate medical education.^42^ As indicated earlier, Lao PDR has one training institution for physicians and, as such, this survey of students effectively incorporates a large share of the students nearing graduation throughout all of Laos.^16^ Table 3 characterizes the medical student sample (n = 329) which included fifth-year students (n = 197), sixth-year students (n = 127), and post-graduate students in the Family Medicine program (n = 5). Students were located either at clinical internships in three provincial hospitals (n = 20 Luang Prabang, n = 25 Savannakhet, n = 20 Champasak) or on site at the University of Health Sciences in Vientiane (n = 264).

**Table 3:** DCE Sample Statistics for Medical Students (n = 329)

**Descriptor**	**Number (%)**
Age	24.7 (5.1)^a^
Gender	
Female	191 (58.1)
Male	138 (41.9)
Rural experience	
Yes	194 (59.0)
No	135 (41.0)
Marital status	
Yes	46 (14.0)
No	283 (86.0)
Have children	
Yes	30 (9.1)
No	299 (91.9)
Prior health worker experience	
Yes	39 (11.9)
No	290 (88.1)
Rural rotation in study	
Yes	263 (79.9)
No	66 (20.1)
Religion	
Buddhist	274 (83.3)
Muslim	44 (13.4)
Christian	8 (2.4)
Other	3 (0.9)
Ethnicity	
Lao	271 (82.4)
Hmong	37 (11.2)
Khmou	8 (2.4)
Other	13 (4.0)
Tuition source	
Family/self	269 (81.8)
Government sponsored	53 (16.1)
NGO sponsored	6 (1.8)
Other	1 (0.3)

Abbreviations: NGO, non-governmental organization; DCE, discrete choice experiment. ^a^Age category lists the mean age and SD in parentheses. Source: Capacity*Plus* Survey (2012).

### Valuation Analysis

In order to calculate the economic value of individual incentive components, we ran a mixed logit regression on the DCE medical student preference data. As the mixed logit requires distributional specifications, we assumed the parameters associated with the incentive components had a random normal distribution^ [1] ^. Given the distributional assumption, the mixed logit regression generates the parameters (mean and standard deviation [SD] of the relative utility of each component) which maximizes the likelihood that the data we see would have occurred.^43^ In order to assess valuation for each component, we divide the mean coefficient for each of the respective incentive components by the mean salary coefficient (at 100% salary level) and then multiply that figure by the reported public sector annual physician compensation (751450 LAK per month or US$1128 per year-2012). We assume that students are aware of this compensation level as they respond to the survey. So for component (c) and salary (s) valuation for a specific component would be expressed as:

(1) $$Valuation_{c}=\frac{\beta_{C}}{\beta_{S}}\times Annual\;Salary$$

Standard errors (SEs) for valuation (or willingness to pay) measures are generated using the delta method – an approach previously utilized and validated in similar DCE analyses.^17,25,44^ DCE analyses are conducted with STATA v. 13 (StataCorp LP, College Station, TX, USA). We also ran a mixed logit using a willingness-to-pay approach (rather than preference approach) in which income is a random rather than fixed parameter and found similar results for the resulting coefficients, SEs, and SDs (Results are reported in the Technical Appendix, Exhibit 3).

### Physician Density and Cost-Effectiveness

In order to evaluate the cost-effectiveness of hypothetic incentive packages (rather than individual components), we generate a model with three basic parts: (1) Estimation of the effect of rural incentive packages on rural and urban distribution of health workers over time relative to rural and urban population sizes, (2) Calculation of the direct costs of each incentive package, and (3) Assessment of the health effects associated with the geographic redistribution of health workers.

#### Estimation of Health Worker Distribution

Given that some incentive package costs are variable and the health effects attributable to each package depend on location decision of health workers, the model first projects the number of health workers in three categories over time—(1) private sector workers [assumed to practice in urban areas: 2011 Lao PDR MoH reported share = 0.30], (2) public sector urban workers [2011 share = 0.54], and (3) public sector rural workers [2011 share = 0.16].^40^ In the model, each of these sub-groups suffers attrition due to mortality, retirement and emigration (base case: 2.5% per year based on prior estimates of health worker production and attrition), but will also experience growth as a result of graduates entering the workforce within each group.^45,46^ Sensitivity analyses test whether the shift in these baseline distributions affects optimal incentive package choice.

In the absence of a rural incentive package, we assume that new entrants will enter private, public-urban and public-rural service at the same rates as the current distribution of health workers for each cadre. If a rural incentive package were instituted on a voluntary basis, we conservatively assume that the share selecting private service will remain at its historic rate, but the allocation of public workers between urban and rural sites will tend toward more rural workers. Specifically, for a particular incentive package (*i*) offered to physicians in time period (*t*) the calculation for the estimate of the new doctor of medicine (MD) graduates entering a rural public position for any particular incentive package is:

New MDs_Rural Public i,t_ = New MDs_t_ * Share MDs_public_ * Share MDs_Rural|Public_ * [2*PPI_i_]

where the PPI_i_ represents the Predicted Preference Impact estimate generated from the mixed logit DCE regression for each incentive package. The preference impact measure estimates what percentage of the sample population would prefer a job posting that offers a position which includes incentives relative to a position that offers the most basic incentive level.^47,48^ A PPI of 0.5 indicates that individuals are indifferent between the current package and a basic package (salary alone). Under this condition we assume that the relative risk of new physicians selecting rural postings equals 1 (or 2*PPI). Also note that PPI has an upper bound value of 1.0 (or 100%). We make the conservative assumption here that the relative risk of new physicians entering the rural postings doubles (RR = 2) under this extreme case. We note this may be a somewhat conservative assumption for two reasons. First, a recent health workforce DCEs in a similar geography (Vietnam) which did explicitly include “rural vs. urban” location as an attribute as well as other attributes similar to the attributes in our Lao PDR study such as continuing education, skills development, facilities/equipment access and housing found that the probability of rural uptake approximately doubled for each of the following incentives: facilities/equipment (2.2 Odds Ratio [OR]), long term education (2.3 OR), skills development (2.1 OR) or home subsidy (1.7 OR).^25^ By assuming a maximum OR of two in our study, even if *all* incentives are implemented, our uptake probabilities are likely biased downward, our cost-effectiveness ratios are likely biased upwards and we are more likely to reject incentive packages as cost-effective. Second, in our model we are narrowly focusing on the expected responses of graduating medical students to voluntary rural incentive packages. If these packages also motivate existing physicians to shift their location preferences the number of physicians in rural positions would increase (For more detail on the location decision estimation process see Technical Appendix, Exhibit 3a). In fact, when we estimate valuations for existing physicians using the same DCE survey (n = 104), we found similar results and valuations among practicing physicians in relation to medical students (see Technical Appendix, Exhibit 6). Another reason for our conservative approach is that, as the case with any DCE, each respondent’s future behavior may deviate (either positively or negatively) from their current expected behavior as preferences may shift over time.

Given the similar valuations placed on each of the attributes relative to salary in the physician and student DCE surveys, we make the simplifying assumption that preferences will stay static over time, although we do conduct univariate sensitivity analyses to see if the optimal incentive program choice changes for individuals with high (+1 SD) or low (-1 SD) weights for attributes which have statistically significant SDs. Other prior work on DCEs have used dynamic models and the richness of mean and SD output from DCEs in student and practicing health worker samples to estimate shifts in location for a cohort of health workers over time with Monte Carlo simulations to generate outcomes measuring rural health worker rural years and total variable costs of incentive programs.^33,34^ In our case, the static assumption of preferences makes attribution of fixed costs (discussed below) across individuals clearer. Two additional limitations deserve mention with respect to the DCE survey. First there was no definition of “rural” provided in the DCE so individuals may interpret the “rural” characterization differently. Second, no formal dominance testing was conducted on the sample.

#### Direct Costs of Incentive Package Components (Fixed and Variable Costs)

The incentive packages include components that result in variable costs that depend on the degree of annual uptake by physicians graduates and fixed costs that do not respond to uptake (Table 4). Future costs are calculated in real terms by multiplying the relevant present value cost by ‘1 + inflation rate’ estimated by the World Economic Outlook Database and dividing by ‘1 + financial discount rate.’ As inflation rates are only estimated for a 5-year window, we used the financial discount rate (base case = 0.03) as the inflation rate after year 5.

**Table 4:** Costs for Rural Incentive Package Components in Lao PDR^a,b^

**Incentive Package Components**		**Incentive Package Components**	
**(Uptake Dependent)**		**(Independent of Uptake)**	
**Housing incentives**		**Facility improvement (district level)**	
Construction (single family home)^c^	$12 500	DH facility Investment (upgrade 1 building)	$100 000
Allowance (annual)	$360	Number of DH facilities upgrades per year^d^	10
**Transport (motorbike)**		DH facility Investment (equipment upgrade)	$25 000
Annual cost of lease	$500	**Facility improvement (local level)**	
Maintenance	$125	HC facility building investment (upgrade)	$25 000
**Additional training**		Number of HC facilities upgrades per year^e^	100
Cost of additional training (MD)^c^	$5600	HC facility equipment investment (upgrade)	$4250
**Career promotion** ^e^			
Career promotion (MD)	$1500		

Abbreviations: PDR‏, People’s Democratic Republic; MD, doctor of medicine. ^a^Source: Capacity*Plus* working paper “Costing of Incentives to Attract and Retain Rural Health Workers in Lao PDR” unless otherwise noted.^49^ ^b^Costs are in $US 2011 unless otherwise specified. Annual discount rates for multiyear costs and benefits are 0.03 for the financial discount rate and 0.03 for health effects (QALY) discount rate. ^c^Note that 18% of qualifying physicians receive additional training in any one year so the average cost across qualifying physicians is $1008. ^d^There are a total of 120 District Level Health Facilities (DH). ^e^There are a total of 836 Local Level Health Facilities (HC).

Fixed cost components include the capital improvement and equipment upgrades for district (n = 120) and local (n = 836) health facilities. These improvements occur over time (10 facilities per year in the case of district facility upgrades; 100 per year in the case of local facility upgrades). As the facility improvements are capital costs, the accounting of costs may follow either a “cash” or “depreciation” accounting method. Under traditional cash accounting, our base case assumption, all costs accrue immediately. In the case of the depreciation approach, we assume a “straightline” 30-year depreciation schedule for capital projects and a 7-year schedule for the equipment acquisitions.

Variable costs include housing construction, housing allowances, transportation (motorcycles), additional training, and accelerated career advancement. Similar to the facility improvement costs, the housing construction costs can either follow a “cash” or “depreciation” accounting methodology. Housing and housing allowances are offered only to *new* rural health workers. Also, in the case of physicians only 50% of the eligible physician pool is assumed to accept the housing construction benefit (as estimated in prior costing studies from Laos). With respect to the transportation benefit, the model assumes 100% uptake by eligible health workers.

Accelerated career promotion results in access to grants for additional education and conferences. Only new health workers qualify for accelerated career promotion (in some cases occurring after 1 year of service, in some cases after 2 years of service). Additional training, a separate benefit, is conferred across all health workers (not just new health workers), but the annual probability of uptake is low (0.18 for MDs) since individuals only qualify periodically.

#### Estimation of Health Effects

We rely on a cross-national WHO analysis that estimates a non-linear relationship between physician density and infant mortality rate (IMR), under-5 mortality rate (excluding infant mortality, U5MR) and maternal mortality rate (MMR) to assess the discounted life years saved as a result of improved health worker density in rural areas (and the *reduction* in discounted life years that occurs due to relatively lower health worker density in urban areas than would be the case in absence of a rural incentive package).^39^ Given we are examining health effects *within* Lao PDR, we rely on the assumption that cross-national effects related to density have similar dynamics within a country. We concentrate on these three outcomes as there is no known reliable data on the effect of other health outcomes but also note that the healthy years contributed by these outcomes affect young cohorts (infants, young children and relatively young mothers) so these outcomes represent more sizable reduction (on a per case basis) than, say, treatment of a chronic condition late in life. We assume that the cross-national estimates serve as reasonable proxies for gauging the effects of within-country physician density variation on health outcomes. While we accept that the relationship between density and health outcomes may suffer from potential endogeneity^ [2] ^, we posit that there is likely a positive causal impact of health workers on health outcomes and this impact likely extends to outcomes outside the three mortality rates focused on in this study. To the extent this is the case, our estimates are likely underreporting the impact of physician density on health. Nevertheless additional research which more precisely delineates the causal effects of density on health outcomes will provide a sharper measure of the health implications in future studies.

Calculation of the discounted life years saved as a result of the rural incentive packages occurs in two steps. In the first major step, we estimate how the annual outcome (IMR, MMR, and U5MR) incidence changes in both rural and urban settings. Specifically for the first step, the model starts by calculating the health worker density in year (t) and location (l, either rural or urban). For the denominator, we use projections and growth rates from the 2011 World Urbanization Prospects report from the United Nations (UN) Economic and Social Affairs Population Division to estimate the population and rural/urban distribution for every year in the analysis.^37^ Health worker densities are generated for both the scenario when the rural incentive package is instituted and the scenario when no package is instituted. We can estimate the percent change in density by location and year and multiply this by the coefficient from the log-log density regression result in the WHO analysis (which reflects the elasticity of change in outcome with respect to the change in density of health workers) for each outcome measure.

(2) $$[Coef_{0}]\times [\%\Delta \;Density_{l,t}]=\%\Delta \;Outcome_{o,l,t}$$

Knowing the baseline IMR, MMR or U5MR incidence for Lao PDR, we then generate an absolute figure for cases averted (or gained in the case of urban health worker densities that decrease as a result of the incentive package) for each outcome in each year. The net number of cases averted (or gained) at the country level is simply the product of the cases averted in rural areas plus the cases gained in urban areas. Thus, for each year of the analysis, we determine the expected number of IMR, MMR, and U5MR cases averted at the country level as a result of the shift in densities mediated by the rural health worker incentive package.

In the second step, the net reduction in country level incidence calculated for each outcome is multiplied by the expected *discounted* QALYs associated with each outcome. For MMR, we assume that mothers are age 25 and estimate life expectancy remaining from that point.^40^ Healthy years are discounted at a 3% rate in the base case. Each of the life years is multiplied by an age-specific QALY/Life Year ratio which results in QALY measure for each year of life by age. Absent age-specific QALY-life year ratios for Laos, we relied on the general population measures reported previously.^50^ The result of the second step is a measure of discounted QALYs saved for each outcome (o) in each year (l) across all of Lao PDR based on the incentive package (i).

Hence given the direct costs and the health benefit estimation, we generate an average cost-effectiveness ratio for each of the 15 incentive packages (relative to no incentive package) and incremental cost-effectiveness ratios which identify the most cost-effective packages.^51,52^

## Results

### Valuation of Incentive Components

Relative to utility associated with salary, each of the components has a statistically significantly positive value (Table 5) with mean valuations ranging between $500 (Continuing Education Benefit) to $188 (Improved Facility Quality). The utility valuations remain similar across student subgroups (all student with the exception of post-graduates [n = 324], students with a rural rotation experience [n = 263], students without a rural rotation experience [n = 66], fifth year students [n = 197], sixth year students [n = 127]; see Technical Appendix Exhibit 5 for regression results). A sample of practicing physicians (n = 104) had relatively similar utilization coefficients although the practicing physicians valued transportation, career promotion and facility quality more and continuing education somewhat less than medical students (see Technical Appendix, Exhibit 6). Naturally these valuations are sensitive to the level of public sector salary (estimated value = $1128) which we assume is consistent over time in the public sector.

**Table 5:** Mixed Logit Regression Results, Implied Valuations (n = 7896 Evaluations Across 329 Medical Students) and Actual Costs

**Incentive Package Component**	**Mean Utility Coefficient** ^a^ ** Estimates** **(SE)**	**SD** **(If Applicable)** ^b^	**Implied Valuation of Component $US** ^c^ **(95% CI** ^d^ **)**	**Annual Cost Estimate ($US)**	**Valuation/Cost** **(95% CI)**
Salary (annual salary = $1128 )	2.46 (0.17)	--	$1128	$1128	1.00
Housing allowance	0.71 (0.07)	0.24	$326 ($260–$392)	$360	0.91 (0.72–1.09)
Housing provision	0.68 (0.06)	0.23	$312 ($249–$375)	$417^e^	0.75 (0.60–0.90)
Career promotion (immediate)	0.67 (0.08)	0.80***	$307 ($231–$382)	$1500	0.20 (0.15–0.25)
Career promotion (1 year wait)	0.46 (0.06)	0.10	$211 ($155–$270)	$1406^f^	0.15 (0.11–0.19)
Continuing education benefit (1 year wait)	1.09 (0.08)	0.59***	$500 ($415–$579)	$945^g^	0.53 (0.44–0.61)
Continuing education benefit (2 year wait)	0.62 (0.06)	0.13	$284 ($219–$348)	$897^g^	0.32 (0.24–0.39)
Facility quality	0.41 (0.06)	0.80***	$188 ($129–$242)	$1030^h^	0.18 (0.12–0.24)
Transportation (official use only)	0.66 (0.06)	0.12	$303 ($233–$369)	$625	0.48 (0.37–0.59)
Transportation (official and informal use)	0.80 (0.07)	0.36**	$367 ($292–$441)	$625	0.59 (0.47–0.70)

Abbreviations: SE, standard error; SD, standard deviation; DCE, discrete choice experiment; PDR‏, People’s Democratic Republic; MoH, Ministry of Health. ^a^ All utility coefficients are significant at the *P* < .01 level. We also estimated the valuations using a willingness to pay DCE model (with salary as a variable rather than fixed parameter estimate) and generated very similar coefficient estimates. ^b^ *P* < .001***, *P* < .01**, *P* < .05* ^c^ Salary estimate for a public sector physician in Lao PRD for 2011-2012 was 751 450 LAK (Lao PDR MoH). The exchange rate value in $US is $1128 (exchange rate = 7993 LAK per $US of 8/1/2012 XE.com). Implied valuations are calculated by dividing the coefficient value for each component by the salary coefficient and multiplying by the annual salary for physicians in Lao PDR. Online technical appendix also shows results for valuations of practicing physicians which were similar to medical students. ^d^ 95% CIs are calculated using the delta method. ^e^ Housing provision cost estimate assumes $12 500 cost is depreciated over a 30-year period. ^f^ Career promotion cost with one year lag is equal to initial cost divided by 1.067 given inflation of 6.7% expected over the first year. ^g^ Continuing education after one and two years are discounted using inflation (0.067 in year one and 0.053 in year two according to World Bank estimates). Also the benefit is conferred to just 18% of the qualifying physician population in any one year. ^h^ Facility quality is a fixed cost so per person variable cost estimates depend on uptake. We use package five to estimate uptake (21 physicians) and calculate total year 1 depreciated costs ($213 K). We then assume that one tenth of the costs are attributable to the rural incentive package as there are likely other benefits which accrue to the health system toward which some of the costs could be allocated (eg, attracting other health workers types, etc). This cost estimate assumes “straight line” depreciation accounting for both district and regional health center capital and equipment expenditures. Capital costs are depreciated using a 30-year schedule and equipment 7 years.

With the exception of housing allowances the ratio of private valuation relative to program costs for incentives were generally significantly less than one and capital intensive attributes (eg, capital expense to improve facility quality) had relatively low valuations in relation to cost. The fact that respondents generally value incentives at a *lower* level than the cost to the government payer may reflect their medical student status but does indicate they are not overestimating the value of components. We interpret this finding as support for DCE methodology as a valid mechanism to assess behavioral response. If anything, the bias appears to tilt *against* changing behaviors in response to programs – the standard critique of DCEs frequently argues otherwise.

While initial costs exceed individual benefit value, several of the benefits of these program components likely yield significant spillovers outside the narrow purpose of motivating physicians to relocate. For example, upgrading facilities naturally will not only motivate more health workers to practice in rural settings, but also will likely improve quality of care and access measures in ways that are external to our analyses. Those components with the highest individual value/program cost ratios are housing allowances and provision and transportation programs – components in which the social benefit is likely concentrated on the qualifying physicians with little additional spillovers. The indirect benefits which accrue to society via improved health as a result of the resultant shifts in physician geographic distribution are not accounted for in these valuation ratios.

### Predicted Preference Impact Estimates of Incentive Packages

The expected predicted preference impacts (PPIs) of the 15 incentive packages ranged from 0.955 to 0.722 (Figure 1). Those packages with more components would naturally would be more preferable and generally have larger PPI values. Of course, the cost of the incentive packages has not yet been factored into the analysis, but this provides reassuring evidence that the respondents are providing rational responses.

$$PPI_{p}=\frac{e^{\beta X_{p}}}{e^{\beta X_{s}}+e^{\beta X_{p}}}$$

where β are coefficients from the mixed logit regression, X_P_ are the values associated with each particular incentive package (including base salary) and X_s_ are the values associated with base salary alone.

### Uptake of Rural Positions by Physicians

Relative to no incentive package, the year 5 (year 30) density of rural physicians per 10 000 rural Lao inhabitants is anticipated to increase by between 10% to 18% (41% to 76%) depending on which incentive package is selected (Table 6). These density measures account for the anticipated urbanization the UN expects among the Lao population during this time. The optimal program (incentive package #4, discussed below) is expected to increase density by 15% (5 years) and 65% (30 years), respectively.

**Table 6:** Rural Physician Counts and Density Estimates by Incentive Package for Lao PDR over Time (Medical Student DCE Survey)

**Incentive Package**	**Base Case Estimates**			
	**2016 Estimated No. of Rural MDs**	**2016 Estimated Rural MD Density** ^1^	**2041 Estimated No. of Rural MDs**	**2041 Estimated Rural MD Density** ^1^
**No Incentive**	300	0.74	703	2.08
1	354	0.87	1235	3.66
2	353	0.87	1229	3.64
3	349	0.86	1185	3.51
4	347	0.86	1162	3.44
5	348	0.86	1173	3.48
6	345	0.85	1143	3.39
7	343	0.85	1127	3.39
8	341	0.84	1107	3.28
9	341	0.84	1106	3.28
10	336	0.83	1055	3.12
11	334	0.82	1038	3.07
12	333	0.82	1028	3.05
13	334	0.82	1038	3.08
14	333	0.82	1032	3.06
15	329	0.81	988	2.93

Abbreviations: PDR, People’s Democratic Republic; DCE, discrete choice experiment; MD, doctor of medicine. ^1^Rural density represents the number of rural physicians per 10 000 rural inhabitants.

### Cost-Effectiveness

**Figure 2:**
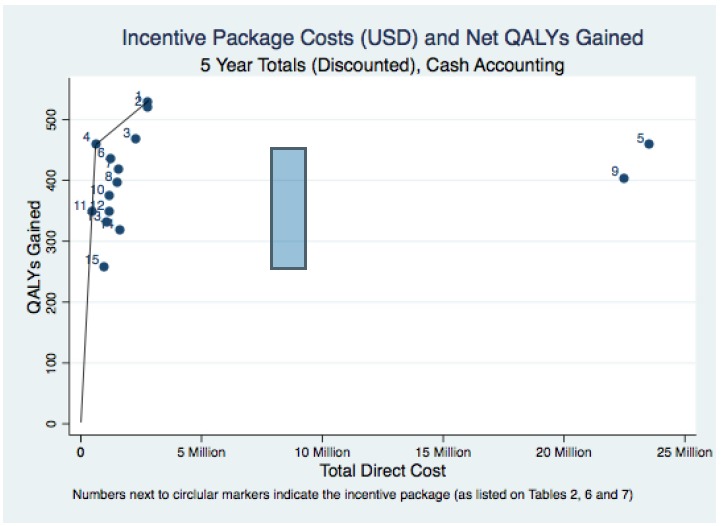
Cost-Effectivensss Frontier Plot for 15 Incentive Packages.

While the valuation and uptake results inform the policy decision regarding voluntary rural incentive programs, the optimal program choice should jointly account for direct program costs and the health benefits that accrue from the program from a societal perspective (Table 7). In the base case (5 years, cash accounting) scenario, average cost per QALY ranged from $1454 (package 4) to $55 762 (package 9) and 7 of the 15 packages had average CERs less than the WHO recommended threshold (GDP*3 = $3786 in Lao PDR). As indicated in Figure 2, two packages (4 and 1) had non-dominated incremental cost-effectiveness ratios (ICERs) and package 4 was the optimal choice as its ICER ($1454) was the only one less than or equal to the WHO threshold CER level.^51,52^

**Table 7:** Rural Incentive Package Cost-Effectiveness Estimates for Laos PDR (Medical Student DCE Survey)

**Incentive Package**	**Base Case** **(Cash Accounting, 5-Year Time Horizon)**			**Base Case** **(Depreciation Accounting, 5-Year Time Horizon)**			**Base Case** **(Cash Accounting, 30 Year Time Horizon)**	
	**Direct Cost of Incentive Package (A) $US (2012)**	**Net Discounted QALYs Gained (B)**	**Average Cost per QALY**	**ICER**	**Average Cost per QALY**	**ICER**	**Average Cost per QALY**	**ICER**
1	$2 836 963	534	$5311	$29 523	$4051	$20 351	$5487	$24 988
2	$2 826 972	528	$5356	Dom.	$4088	Dom.	$5525	Dom.
3	$2 347 845	484	$4851	Dom.	$3523	Dom.	$5150	Dom.
4	$669 983	461	$1454	$1454	$1454	$1454	$2380	$2380
5	$23 594 809	472	$50 022	Dom.	$10 481	Dom.	$9965	Dom.
6	$1 283 630	441	$2909	Dom.	$1511	Dom.	$3083	Dom.
7	$1 604 154	426	$3769	Dom.	$3769	Dom.	$4530	Dom.
8	$1 594 078	406	$3927	Dom.	$3927	Dom.	$4676	Dom.
9	$22 543 898	404	$55 762	Dom.	$9629	Dom.	$8838	Dom.
10	$1 252 563	353	$3550	Dom.	$3550	Dom.	$5092	Dom.
11	$536 504	336	$1598	Dom.	$1598	Dom.	$2416	Dom.
12	$1 239 995	326	$3803	Dom.	$3803	Dom.	$5385	Dom.
13	$1 114 205	337	$3310	Dom.	$3310	Dom.	$4135	Dom.
14	$1 670 451	331	$5054	Dom.	$3390	Dom.	$4957	Dom.
15	$1 047 792	286	$3662	Dom.	$3662	Dom.	$4159	Dom.

Abbreviations: PDR, People’s Democratic Republic; DCE, discrete choice experiment; QALY, quality-adjusted life year; ICER, incremental cost-effectiveness ratio; Dom. = Dominated (Incentive Package is either weakly or strongly dominated by other options).

Across all univariate sensitivity analyses, the average and incremental CERs typically shifted, but the optimal choice remained package 4. The sensitivity analyses included accounting methodology (depreciation vs. cash), duration (30 vs. 5 years), utility value for career promotion (+/- 1 SD), utility value for continuing education benefit (+/- 1 SD), utility value for upgraded facilities (+/-1 SD), utility value for full transportation benefit (+/-1 SD), 50% increase in base salary (new rural physicians only), 50% increase in base salary (apply to all rural physicians), 50% larger (or smaller) initial rural share of physicians, high discount rate (5% vs. 3%), low discount rate (1% vs. 3%), high attrition rate (5% vs. 2.5%), low attrition rate (1% vs. 2.5%), high public sector share of physicians (85% vs. 70%), and low public sector share of physicians (55% vs. 70%). [See Technical Appendix Exhibit 4 for sensitivity analysis result details]. Since package 4 featured non-capital intensive attributes (housing allowance, transportation benefit and accelerated career promotion) its base case 5-year costs were second lowest ($670 K), but its anticipated health effects were fourth highest (461 QALYs gained) among the 15 options.

## Discussion

As the use of DCEs has grown in health economics and health services research, an emergent literature has focused on the job choice of health workers in low- and middle-income countries (LMICs).^19,23-25,53^ Some analyses are now combining program cost data with DCEs to account for the anticipated cost of influencing health worker job choices within these resource constrained settings over time.^33,34^ Our analysis contributes to this literature in the context of Lao PDR and we apply novel methods which can help inform future studies that aim to identify optimal incentive options by accounting for costs and benefits to the health system and improvements in health outcomes. In order to place our results in context it is important to understand the three important caveats of our study: lack of a rural-urban attribute, the assumption of static preferences over time and the association between health worker density and health outcomes.

Given the context of the initial study was focused on rural employment alone, our initial design did not incorporate a rural vs. urban attribute. For future studies in settings with rural disparities, this is an easy adjustment to incorporate in the design phase of a project (albeit sometimes addition of an extra attribute comes at the expense of omitting competing attributes or greater survey complexity). In our case, we estimate the uptake probabilities of rural service indirectly and, as such, adopt a methodology which likely results in more conservative assumptions with respect to uptake. So long as this bias affects our evaluation of incentive packages in a consistent manner, our optimal choice (package 4) likely would still be chosen had the initial design incorporated a geography attribute. Optimally the design would offer an “opt out” option to limit upward uptake bias particularly for nurse or other health worker cadres where the likelihood of a non-healthcare job is somewhat greater than among physicians (in our case it is unlikely that medical students would opt for a non-physician position in the labor market given their monetary and temporal investment).

Given that some of our models focus on a thirty year period (in part to match the depreciation schedules common for cost accounting of major capital expenditures) consideration should be given to how preferences shift over time. In other studies, novel Monte Carlo models with multiple DCE sub-groups at different career stages explicitly allow for the shift in preferences as a cohort ages and account for variability in preference within a particular time period. We assumed that preferences remain static with respect to attributes in the DCE, in part, as the relative utility (in relation to salary) appeared to not deviate too extensively in our medical student sample and our practicing physicians sample.

The last critical caveat for our study is the assumption that the health worker density-health outcomes association is not endogenous. In order to map out the health outcomes impact of health worker redistribution, further study to understand the causal impact of density disparities and health outcomes, particularly *within* countries, would improve predictions for cost-effectiveness approaches. For our Lao PDR study we think we made relatively conservative approach to estimating QALYs since we focus on just a subset of health outcomes. Again, so long as the bias is consistent across attributes, we likely have not deviated from the optimal choice. To the extent the relationship can be clarified in the future, generating cost per QALY CERs may allow for additional comparison with other competing interventions (eg, mandated rural health worker policies, vaccines) vying for resources. One of the benefits of moving health worker DCEs into a cost-effectiveness framework is the ability to not only determine what the optimal choice is for one’s limited study, but also to assess whether that choice represents a wise use of resources relative to a wide range of potential investments on health amid the limited MoH budgets in LMICs.

## Conclusion

Conditional on selecting one of the 15 incentive bundles, the optimal choice from the Lao PDR MoH perspective is package 4 as it combines low cost, non-capital intensive incentives that are effective in motivating graduates to enter into rural service. Our evaluation method builds on prior DCE health worker studies as it assess valuation of attributes and predicts resultant health worker geographic decisions, but we also extend our analysis to incorporate a cost-effectiveness framework that can determine whether the intervention represents a wise use of resources from a cost per QALY perspective. Given the methodological caveats of our study, we believe it is possible we have overestimated the CERs of each of the incentive bundles, but the optimal choice was unlikely to shift among the 15 options as a result of the bias given its superiority relative to the other 14 options. As our optimal option still has an ICER less than the WHO threshold recommendation, we think it represented a judicious choice, but MoHs are certainly not bound to implement the policy. In fact, the Lao PDR MoH opted for a three year mandatory service program for recent health worker graduates—an option which is likely lower in cost, but also may hinder long run rural uptake if incentives are completely excluded. While less expensive, the restrictions of a mandatory program are prompting the Lao MoH to consider inclusion of limited additional benefits perhaps to encourage participants to remain in rural settings after the mandatory window expires. As other countries grapple with how to rebalance the geographic distribution of health workers, they will have to consider whether additional benefits are administratively feasible and which combination of incentives offers the best return on investment within a fixed budget. We encourage further research combining DCEs with cost data to generate more awareness of costs and benefits of health worker incentives programs in LMICs. Whether the novel methods generate cost-effectiveness ratios or other alternative metrics for decision-making, additional research in other geographies that use novel methods will continue to improve the information upon which health worker policies are implemented in resource constrained countries.

## Acknowledgments

My co-authors and I wish to acknowledge USAID as the surveys, data and funding which helped contribute to this research project were provided via support from USAID [USAID Grant Number: AID-GPO-A-00-9-00006]

## Ethical issues

The DCE survey was approved by the Lao PDR MoH National Ethics Committee for Health Research. All analytical work was conducted on de-identified datasets.

## Competing interests

Authors declare that they have no competing interests.

## Authors’ contributions

EK, WJ, and KaT made substantial contributions to conception and design of the research. WJ, KaT, and KhT were involved in the acquisition of data. All authors were involved in analysis and interpretation of data. EK and WJ drafting the initial manuscript and all authors revising it critically for important intellectual content. All authors have given final approval of the version to be published. All authors agree to be accountable for all aspects of the work in ensuring that questions related to the accuracy or integrity of any part of the work are appropriately investigated and resolved.

## Endnotes


[1] Although salary was fixed in our base case, we also ran a specification in which salary was randomly distributed. Valuation results did not vary substantially (see online technical appendix for regression output).

[2] Although to the extent the endogeneity is a result of physicians “selecting” healthier urban populations is likely less prevalent in cross-national analyses since emigration from a country is likely more difficult than emigration from within a country from a rural to urban area.


## Authors’ affiliations

^1^Health Finance & Access Initiative, Bryn Mawr, PA, USA. ^2^IntraHealth International, Washington, DC, USA. ^3^Ministry of Health (Lao PDR), Vientiane, Lao PDR.

## Supplementary Files

Supplementary file 1 contains the technical appendix.

## Key messages

**Implications for policy makers**

- Policy-makers in low- and middle-income countries (LMICs) can combine discrete choice and cost surveys to evaluate the cost-effectiveness of health workforce rural incentive programs.
- In the Lao People’s Democratic Republic (PDR) context, the capital intensive incentives tended to be more expensive relative to the potential benefit they conferred to improve health.
- Investing in better data collection on the link between health outcomes and health worker density and understanding how incentives affect existing health workers will improve the precision of future results.
- Policy-makers should also compare the expected costs and benefits of mandatory rural service programs for recent graduates with voluntary incentive approaches

**Implications for public**

This research identifies the optimal set of incentives to encourage recent physician graduates to voluntarily practice in rural settings upon graduation in Lao People’s Democratic Republic (PDR). These programs will increase the density of health workers in rural settings and improve access to care and health outcomes for rural inhabitants. By selecting the optimal program we also limit the cost to the Ministry of Health (MoH) (and therefore the public).
