# Supplementary material for: Cost-Effectiveness of Rural Incentive Packages for Graduating Medical Students in Lao PDR
Source: Int J Health Policy Manag. 2016 Oct 29;6(7):383–94. doi: 10.15171/ijhpm.2016.141 (PMC5505108; doi:10.15171/ijhpm.2016.141)
Supplement: Supplementary file 1 — contains the technical appendix. [file ijhpm-6-383-s001.pdf]

IJHPM
International Journal of Health Policy and Management

Technical Appendix

Exhibit 1 – DCE Survey for Medical Students

Understanding health worker preferences for development of a national strategy to increase health worker attraction and retention in rural areas of Lao PDR

Student Cadre: MD Student

Questionnaire Version: _____ **Respondent ID: _______**

Date: ____

Name of School/Hospital: ____

Name of Data Collector: ____

Introduction to the Survey:
Thank you very much for coming today. We are working with the Ministry of Health to learn about the factors that motivate health care providers to work in facilities located in rural areas. As a student looking towards the future, we are interested in knowing more about what incentives or characteristics about a job would influence your decision to work in a rural area. This survey should take approximately 20-30 minutes.

You will be asked questions to obtain demographic and other background information. Then, there will be a series of questions about hypothetical job postings. Your participation will help us quantify the attractive power of incentives and characteristics to motivate health workers to accept postings in rural areas.

Please give your most honest responses throughout the questionnaire. There is no right or wrong answer. This information will assist the Ministry of Health to develop a national strategy to increase health worker attraction and retention in rural areas.

If you have any questions during the survey, please feel free to ask me.

Thank you for your participation!

Section A: Demographic and Background Information

First, we will ask you a set of questions about your personal background. Please note that the numbers in parentheses next to the boxes have no meaning for your response. They are used only as coding for data entry.

1. In which university are you currently enrolled?

University of Health Sciences (Vientiane) ☐ (0)
Other (specify)________________________ ☐ (1)

2. In which year of study are you?

6th year ☐ (0)
5th year ☐ (1)
Other (specify)___________ ☐ (2)

3. In which program are you?

Direct entry ☐ (0)
Upgrading ☐ (1)

4. What is your gender?

Male ☐ (0)
Female ☐ (1)

5. What is your age, in years?

_______ years

6. From the age of 6 onwards, have you at any time lived in a rural area for more than 1 year at a time?

No ☐ (0)
Yes ☐ (1)

7. What is your marital status?

Single ☐ (0)
Married and live with my spouse ☐ (1)
Married but DO NOT live with my spouse ☐ (2)

8. How many living children do you have? (if none, enter 0)

____ children

9. What is your religion?

Buddhist ☐ (0)
Christian ☐ (1)
Muslim ☐ (2)
Animist ☐ (3)
Other (specify)________________________ ☐ (4)
No religion ☐ (5)

10. To which ethnic group do you belong?

Lao ☐ (0)
Hmong ☐ (1)
Khmou ☐ (2)
Other (specify) ___________________ ☐ (3)

Section B: Professional background
Now we are going to ask you a few questions related to your school and work experience.

11. How do you pay tuition for your current study program?

I am sponsored by the Government of Laos ☐ (0)
I am sponsored by a non-governmental organization ☐ (1)
I pay my tuition fee myself or with help from family ☐ (2)
Other (specify) ____ ☐ (3)

12. Before enrolling in your current study program, how many years of work experience did you have as a health worker (if none, enter 0)?

_____years

13. Have you at any time worked as a health worker in a rural area for more than 6 months at a time

No ☐ (0) SKIP to Question 15.
Yes ☐ (1)

14. Please rate your experience overall working in a rural area.

Excellent ☐ (0)
Very Good ☐ (1)
Good ☐ (2)
Fair ☐ (3)
Poor ☐ (4)

15. Are you under any obligation or have you made any commitment to work in a rural area after graduation?

No ☐ (0)
Yes ☐ (1)

16. During your studies thus far, have you done service in a rural area as part of your study program?

No ☐ (0) SKIP to Question 19.
Yes ☐ (1)

17. How many weeks did you spend doing service in a rural area during your study program?

______weeks

18. Please rate your experience overall doing service in a rural area during your study program.

Excellent ☐ (0)
Very Good ☐ (1)
Good ☐ (2)
Fair ☐ (3)
Poor ☐ (4)

19. Please rate how likely you are to work in a rural area at some point in the future?

Very unlikely ☐ (0)
Unlikely ☐ (1)
Likely ☐ (2)
Very likely ☐ (3)

20. Thinking in general about the possibility of working in a rural area, which of the following is the **MOST IMPORTANT** factor in your decision to work in a rural area (**select ONLY one**)?

The facility has sufficient staff type and number and equipment is always available/working, according to facility type ☐ (0)

You have accelerated career promotion/advancement opportunities ☐ (1)

You are provided housing ☐ (2)

You are provided a higher salary ☐ (3)

You qualify for further study and a scholarship ☐ (4)

You are provided transport for official and personal use ☐ (5)

Other (specify)____ ☐ (6)

21. The government of Laos is thinking of providing incentives and other motivational factors (which could include increased salary, housing, further study, better quality facilities) to encourage health workers to work in rural areas. If such incentives were available, would you consider working in a rural area?

No ☐ (0)
Yes ☐ (1)

Section C: Job Scenarios

Imagine that you have just successfully completed your study program. You are looking for jobs in the newspaper, radio, and other sources, and find that there are two advertised job postings in government health facilities. Both of the facilities described in the job postings are located in rural areas. Both facilities are of equal distance from the nearest big town and are of equal distance from the capital city. However, each of the two postings provides different characteristics or benefits.

Please imagine yourself in this situation and make a decision as to which of the two presented postings you would prefer. For the sake of this survey please assume that you would indeed receive the full benefits described for the postings. In making your choice, please carefully read the full list of benefits for each posting and do not imagine any additional features of the postings.

There are 12 different scenarios presented. Please note that while they may look similar at a quick glance, they are indeed each very different.

Attributes and Levels: MD/Medical Students

Attribute	Levels
**Quality of the facility ("quality")**	1. Insufficient staff type and number, equipment NOT always available/working, according to facility type<br>2. Sufficient staff type and number; equipment always available/working, according to facility type
**Career promotion ("career")**	1. Promoted to permanent staff after 2 years<br>2. Promoted to permanent staff after 1 year<br>3. Directly promoted to permanent staff upon posting
**Housing**	1. No housing provision<br>2. Housing allowance provided<br>3. Provide dormitory/housing
**Salary**	1. No additional salary<br>2. 30% additional salary<br>3. 40% additional salary<br>4. 50% additional salary
**Continued Education ("conted")**	1. Qualify for further study and scholarship after 3 years<br>2. Qualify for further study and scholarship after 2 years<br>3. Qualify for further study and scholarship after 1 year
**Transport**	1. No transport provided<br>2. Transport provided for official activity/routine work<br>3. Transport provided for official and personal use

Example Job Scenario Pair Question from DCE Survey – Medical Students and Physicians

Which of these two job postings do you prefer? Select one by marking the circle under the job posting you prefer.

	Job A	Job B
Quality of the Facility	Insufficient staff type and number, equipment NOT always available/working, according to facility type	Sufficient staff type and number; equipment always available/working, according to facility type
Career Promotion	Directly promoted to permanent staff upon posting	Promoted to permanent staff after 1 year
Housing	Housing allowance provided	No housing provision
Salary	No additional salary	30% additional salary
Continued Education	Qualify for further study and scholarship after 3 years	Qualify for further study and scholarship after 1 year
Transport	No transport provided	Transport provided for official activity/routine work
	○	○

Exhibit 2: Efficiency of Survey Design

CBC Design Efficiency Test
Task generation method is 'Complete Enumeration' using a seed of 1.
Based on 5 version(s).
Includes 60 total choice tasks (12 per version).
Each choice task includes 2 concepts and 6 attributes.

A Priori Estimates of Standard Errors for Attribute Levels

Att/Lev		Freq.	Actual	Ideal	Effic.	
1	1	60	(this level has been deleted)			Insufficient staff type and number and equ;ipment NOT always available for facility type/level
1	2	60	0.1832	0.1826	0.9934	Sufficient staff type and number and equipment always available for facility type
2	1	40	(this level has been deleted)			Promoted to permanent staff after 2 years
2	2	40	0.2708	0.2582	0.9092	Promoted to permanent staff after 1 year
2	3	40	0.2613	0.2582	0.9761	Directly promoted to permanent staff upon posting in rural/remote facility
3	1	40	(this level has been deleted)			No housing provided
3	2	40	0.2598	0.2582	0.9879	Housing allowance provided
3	3	40	0.2616	0.2582	0.9741	Dormitory/housing provided
4	1	30	(this level has been deleted)			No additional salary
4	2	30	0.3225	0.3162	0.9612	30% additional salary
4	3	30	0.3218	0.3162	0.9655	40% additional salary
4	4	30	0.3285	0.3162	0.9264	50% additional salary
5	1	40	(this level has been deleted)			Qualify for further study and financial support after 3 years in rural facility
5	2	40	0.2621	0.2582	0.9706	Qualify for further study and financial support after 2 years in rural facility
5	3	40	0.2622	0.2582	0.9695	Qualify for further study and financial support after 1 year in rural facility
6	1	40	(this level has been deleted)			No transport provided
6	2	40	0.2639	0.2582	0.9571	Transport provided for official activity
6	3	40	0.2645	0.2582	0.9533	Transport provided for official and personal use

Note: The efficiencies reported above for this design assume an equal number of respondents complete each version.

Two-Way Frequencies

Att/Lev	1/1	1/2	2/1	2/2	2/3	3/1	3/2	3/3	4/1	4/2	4/3	4/4	5/1	5/2	5/3	6/1	6/2	6/3
**1/1**	60	0	20	21	19	19	20	21	15	15	15	15	20	20	20	21	19	20
**1/2**	0	60	20	19	21	21	20	19	15	15	15	15	20	20	20	19	21	20
**2/1**	20	20	40	0	0	13	14	13	10	10	9	11	13	13	14	13	13	14
**2/2**	21	19	0	40	0	13	14	13	10	11	11	8	15	13	12	15	13	12
**2/3**	19	21	0	0	40	14	12	14	10	9	10	11	12	14	14	12	14	14
**3/1**	19	21	13	13	14	40	0	0	10	10	10	10	15	13	12	14	13	13
**3/2**	20	20	14	14	12	0	40	0	10	10	10	10	12	13	15	12	14	14
**3/3**	21	19	13	13	14	0	0	40	10	10	10	10	13	14	13	14	13	13
**4/1**	15	15	10	10	10	10	10	10	30	0	0	0	10	10	10	10	11	9
**4/2**	15	15	10	11	9	10	10	10	0	30	0	0	11	9	10	10	10	10
**4/3**	15	15	9	11	10	10	10	10	0	0	30	0	9	12	9	11	9	10
**4/4**	15	15	11	8	11	10	10	10	0	0	0	30	10	9	11	9	10	11
**5/1**	20	20	13	15	12	15	12	13	10	11	9	10	40	0	0	13	13	14
**5/2**	20	20	13	13	14	13	13	14	10	9	12	9	0	40	0	14	13	13
**5/3**	20	20	14	12	14	12	15	13	10	10	9	11	0	0	40	13	14	13
**6/1**	21	19	13	15	12	14	12	14	10	10	11	9	13	14	13	40	0	0
**6/2**	19	21	13	13	14	13	14	13	11	10	9	10	13	13	14	0	40	0
**6/3**	20	20	14	12	14	13	14	13	9	10	10	11	14	13	13	0	0	40

Exhibit 3: DCE Summary Results – Mixed Logit and WTP Regression Estimates

Incentive Package Component	Standard Mixed Logit Regression: Mean Utility Coefficient[1] Estimates (Standard Error)	Standard Mixed Logit Regression: Standard Deviation[2]	Standard Mixed Logit Regression: Implied Valuation of Component $US[3] (95% CI[4])	WTP Regression: Mean Coefficient Estimates (Standard Error)[5]	WTP Regression: Standard Deviation[2]	WTP Regression: Implied Valuation of Component $US[3] (95% CI[4])
Salary (Annual Salary= $1128)	2.46 (0.17)	--	$1,128	0.97 (0.08)	0.67***	$1096 ($924-$1269)
Housing Allowance	0.71 (.07)	0.24	$326 ($260-$392)	0.29 (0.03)	0.05	$327 ($264-$389)
Housing Provision	0.68 (.06)	0.23	$312 ($248-$375)	0.27 (0.03)	0.08	$301 ($241-$361)
Career Promotion (Immediate)	0.67 (.08)	0.80***	$307 ($231-$382)	0.29 (0.02)	0.26***	$324 ($256-$392)
Career Promotion (1 Year Wait)	0.46 (.06)	0.10	$213 ($155-$270)	0.20 (0.03)	0.01	$223 ($169-$276)
Continuing Education Benefit (1 Year Wait)	1.09 (.08)	0.59***	$497 ($415-$579)	0.45 (0.03)	0.20***	$510 ($434-$587)
Continuing Education Benefit (2 Year Wait)	0.62 (.06)	0.13	$284 ($219-$348)	0.25 (0.03)	0.01	$279 ($218-$340)
Facility Quality	0.41(.06)	0.80***	$185 ($129-$242)	0.17 (0.02)	0.29***	$193 ($140-$248)
Transportation (Official Use Only)	0.66 (.06)	0.12	$301 ($233-$369)	0.26 (0.03)	0.03	$297 ($234-$360)
Transportation (Official and Informal Use)	0.80 (.07)	0.36***	$366 ($292-$441)	0.33 (0.08)	0.03	$375 ($302-$448)

[1]All utility coefficients are significant at the $P < .01$ level. We also estimated the valuations using a willingness to pay DCE model (with salary as a variable rather than fixed parameter estimate) and generated very similar coefficient estimates (relative to the salary coefficient).

[2] P <.001***, P <.01**, P <.05*

[3]Salary estimate for a public sector physician in Lao PRD for 2011-2012 was 751,450 LAK (W. Jaskiewicz from MoH Laos). The exchange rate value in $US is $1128 (exchange rate = 7993 LAK per $US of 8/1/2012 XE.com). Implied valuations are calculated by dividing the coefficient value for each component by the salary coefficient and multiplying by the annual salary for physicians in Lao PDR.

[4]95% CIs are calculated using the delta method

[5]WTP regression coefficients are divided by salary

```
Mixed logit model                                 Number of obs   =       7896
                                                  LR chi2(9)      =     154.97
Log likelihood = -2139.5089                       Prob > chi2     =     0.0000

------------------------------------------------------------------------------
      choice |      Coef.   Std. Err.      z    P>|z|     [95% Conf. Interval]
-------------+----------------------------------------------------------------
Mean         |
    salary_a |   2.464348   .1651824    14.92   0.000     2.140597      2.7881
 house_allow |   .7128856   .0653482    10.91   0.000     .5848055    .8409658
  house_prov |   .6812241   .0644884    10.56   0.000     .5548291    .8076191
 career_pro0 |   .6697503    .075209     8.91   0.000     .5223434    .8171572
 career_pro1 |   .4643231   .0609779     7.61   0.000     .3448086    .5838376
     coned_1 |   1.085816   .0757079    14.34   0.000     .9374313    1.234201
     coned_2 |    .619682   .0619217    10.01   0.000     .4983177    .7410463
   quality_a |   .4051129   .0615178     6.59   0.000     .2845403    .5256856
   trans_off |   .6583239    .063668    10.34   0.000      .533537    .7831109
  trans_both |   .8001244   .0672841    11.89   0.000     .6682499    .9319989
-------------+----------------------------------------------------------------
SD           |
 house_allow |  -.2387377   .1299566    -1.84   0.066    -.4934479    .0159725
  house_prov |   .2281767   .1768794     1.29   0.197    -.1185006     .574854
 career_pro0 |    .802309   .0843734     9.51   0.000     .6369403    .9676778
 career_pro1 |  -.0966943   .1132604    -0.85   0.393    -.3186806    .1252921
     coned_1 |   .5939945     .09756     6.09   0.000     .4027804    .7852086
     coned_2 |   .1345518   .1304097     1.03   0.302    -.1210464    .3901501
   quality_a |   .8001923   .0683078    11.71   0.000     .6663115    .9340731
   trans_off |   .1216367   .1055813     1.15   0.249    -.0852988    .3285722
  trans_both |   .3676548   .1082969     3.39   0.001     .1553968    .5799127
------------------------------------------------------------------------------
The sign of the estimated standard deviations is irrelevant: interpret them as
being positive
```

Mixed logit model in WTP space

Number of obs = 7896
Wald chi2(10) = 1011.50
Log likelihood = -2120.6724
Prob > chi2 = 0.0000

(Std. Err. adjusted for clustering on respond_id)

choice	Coef.	Std. Err.	z	P>|z|	[95% Conf.	Interval]
Mean						
house_allow	.2895774	.0282738	10.24	0.000	.2341616	.3449931
house_prov	.2671849	.0271224	9.85	0.000	.2140261	.3203438
career_pro0	.2868711	.0307724	9.32	0.000	.2265583	.3471839
career_pro1	.1972559	.0243044	8.12	0.000	.1496201	.2448917
coned_1	.4524279	.0346386	13.06	0.000	.3845374	.5203184
coned_2	.2472264	.0274603	9.00	0.000	.1934052	.3010476
quality_a	.1718597	.0242775	7.08	0.000	.1242767	.2194427
trans_off	.2630768	.0283368	9.28	0.000	.2075377	.3186159
trans_both	.3324841	.0330573	10.06	0.000	.2676929	.3972752
salary_a	.9721632	.0781155	12.45	0.000	.8190596	1.125267
SD						
house_allow	-.0524356	.0588915	-0.89	0.373	-.1678608	.0629896
house_prov	.0778959	.0531791	1.46	0.143	-.0263332	.1821251
career_pro0	.2568668	.0374131	6.87	0.000	.1835385	.330195
career_pro1	-.0097885	.0419344	-0.23	0.815	-.0919785	.0724015
coned_1	.2009222	.037886	5.30	0.000	.1266671	.2751774
coned_2	.0108679	.0429343	0.25	0.800	-.0732819	.0950176
quality_a	.298036	.0319126	9.34	0.000	.2354884	.3605836
trans_off	.0298822	.0488995	0.61	0.541	-.0659591	.1257236
trans_both	.0336066	.0855032	0.39	0.694	-.1339766	.2011898
salary_a	.6690052	.0950197	7.04	0.000	.48277	.8552404

The sign of the estimated standard deviations is irrelevant: interpret them as being positive

Exhibit 3a: Details on Estimation of Physician Location Decision

As the survey was initially designed to examine preferences *assuming* a rural position (all positions labeled as 'rural'), geography (urban vs. rural) was not an explicit attribute in the survey. As such, we were *not* able to directly estimate the change in probability associated with accepting a rural position (relative to an urban position) from the beta coefficients in the standard manner where:

$P_{in} = \frac{\exp(\beta' X_{in})}{\sum_{j \in C_n} \exp(\beta' X_{jn})}$ (Ryan et al. p.25).[1]

In order to generate our probabilities, we first examined a DCE from a similar geography (Vietnam) and sample (Physicians) which *did* include rural/urban geographic designations as a attribute and which examined other attributes similar to the attributes in our Lao PDR study such as continuing education, housing etc. (Vujicic, 2013).[2] Below are the DCE results from the Vietnam Study.

**Attribute (Vujicic et al, 2013)**	**Mixed Logit Coefficient**	**Affect on Rural Probability**[1]	**Affect on Odds Ratio**[2]
Official Income (Per 1Mill. VND)	0.149		
Location (Urban = 1)	1.113	--	--
Equipment (Advanced = 1)	0.784	+7.3%	2.2
Long Term Education (Yes = 1)	0.817	+7.7%	2.3
Skills Development (Yes = 1)	0.748	+6.8%	2.1
House Subsidy (Yes = 1)	0.5	+4.1%	1.7
Job A Constant	-0.654	--	--

[1] This reflects the effect of adding the incentive to the probability of accepting a rural position (relative to urban job with equipment and long term education benefit; baseline rural only estimate 7%. Note: baseline rural official salary 4.3M VND, baseline urban salary 3.3M VND. Unofficial salaries tend to be higher in urban areas.)
[2] Odds ratio relative to baseline probability.

Note that typically adding just one of these incentives doubles the probability (and odds) of choosing a rural position in the case of the Vietnam. With respect to our Lao PDR study, we conservatively assumed that the *maximum* increase in the relative risk of selecting a rural position would be 2x the baseline relative risk. We felt this reasonable, since the Lao PDR and Vietnam

studies differ. This more conservative approach likely means that our cost effectiveness ratios are biased upwards and we are more likely to reject incentive packages as cost effective.

Specifically for each of the 15 incentive packages we estimated the predicted preference impact (PPI) which represents the probability of accepting the incentive package (relative to salary alone) $PPI_p = \frac{e^{\beta X_p}}{e^{\beta X_s} + e^{\beta X_p}}$ where β are coefficients from the mixed logit regression, X_P are the values associated with each particular incentive package (including base salary) and Xs are the values associated with base salary alone.

The preference impact measure estimates what percentage of the sample population would prefer a job posting that offers a position which includes incentives relative to a position that offers the most basic incentive level.[3,4] A PPI of 0.5 indicates that individuals are indifferent between the current package and a basic package (salary alone). Under this condition we assume that the relative risk of new physicians selecting rural postings equals 1 (or 2*PPI). Also note that PPI has an upper bound value of 1.0 (or 100%). We make the conservative assumption here that the probability of new physicians entering the rural postings at most doubles (Rel Risk = 2) under the best case.

Armed with the new relative risks we map out the number of students who enter the rural workforce voluntarily under each of the 15 incentive packages. Twenty-three percent public sector MDs are assumed to enter without any incentive (the current share of public sector MDs in rural areas). We multiply this by the relative risk to estimate the number that will enter under any particular incentive package. Note we assume the share of students that enter the public sector is proportional to the current share of public sector MDs (70%) and that this remains fixed over time.

Public sector share and others key assumptions are challenged in the sensitivity analyses for the model.

Exhibit 3b: QALY estimation

Each of the life years are multiplied by an age-specific QALY/Life Year ratio which results in QALY measure for each year of life by age. Absent age specific QALY-life year ratios for Laos, we relied on the general population measures reported by Claxton et al (NICE 2013).[5] These are similar to other population level measures found (Fryback et al, 2007).[6] We did find results from an EQ-5D survey in a similar geography (Vietnam), but it focused on the elderly (60+) population (Hoi et al 2009, Hoi et al 2010).[7,8] The elderly QALY/LY ratios were similar to the UK and US data for older ages (if anything a little higher, biasing our results conservatively downward). Specifically we used the following QALY/LY rations for the following age bands:

**Age**	**QALY/LY Ratio by Age**
0-20	0.95
20-40	0.92
40-60	0.85
60-80	0.76

These QALYs for each year are then discounted to estimate the QALYS associated with saving a life for the three outcome measures for which we have data (IMR, U5M, MM). Each of outcomes has a different life expectancies and different assumed age of the individual: Infant mortality (Laos life expectancy at 0 is 61.3), Under Five Mortality (Life expectancy at 2.5 years old is 65.2 in Laos) and Maternal Mortality (Female Life expectancy at age 22 Laos is an additional 49.3 years). Hence, the QALYs associated for each type of outcome are:

$$QALY_{IMR} = \sum_{i=0,t=0}^{i=61.3} \frac{QR_i}{(1+r)^t} = 26.7\ QALYs\ @3\%\ discount\ rate$$

$$QALY_{U5M} = \sum_{i=2.5,t=0}^{i=65.2} \frac{QR_i}{(1+r)^t} = 26.2\ QALYs\ @3\%\ discount\ rate$$

$$QALY_{MM} = \sum_{i=22,t=0}^{i=71.3} \frac{QR_i}{(1+r)^t} = 23.3\ QALYs\ @3\%\ discount\ rate$$

For IMR, U5M and MM events averted in the future we discount the those back to the base year. So, for example, the current value of saving an IMR in year 5 of our model would be:

$$QALY_{IMR\ yr5} = \frac{QALY_{IMR}}{(1+r)^5} = 23.0\ @\ 3\%\ discount\ rate$$

Exhibit 4: Cost Effectiveness of Incentive Packages – Sensitivity Analysis A
(Standard Deviation Analyses)

Base Case: 5 year/cash accounting

Package	Total Direct Cost	QALYs (5 Years)	Cost per QALY (5 Year)	ICER
1	$2,836,963	534	$5,311	$29,523
2	$2,826,972	528	$5,356	Dom.
3	$2,347,845	484	$4,851	Dom.
4	$669,983	461	$1,454	$1,454
5	$23,594,809	472	$50,022	Dom.
6	$1,283,630	441	$2,909	Dom.
7	$1,604,154	426	$3,769	Dom.
8	$1,594,078	406	$3,927	Dom.
9	$22,543,898	404	$55,762	Dom.
10	$1,252,563	353	$3,550	Dom.
11	$536,504	336	$1,598	Dom.
12	$1,239,995	326	$3,803	Dom.
13	$1,114,205	337	$3,310	Dom.
14	$1,670,451	331	$5,054	Dom.
15	$1,047,792	286	$3,662	Dom.

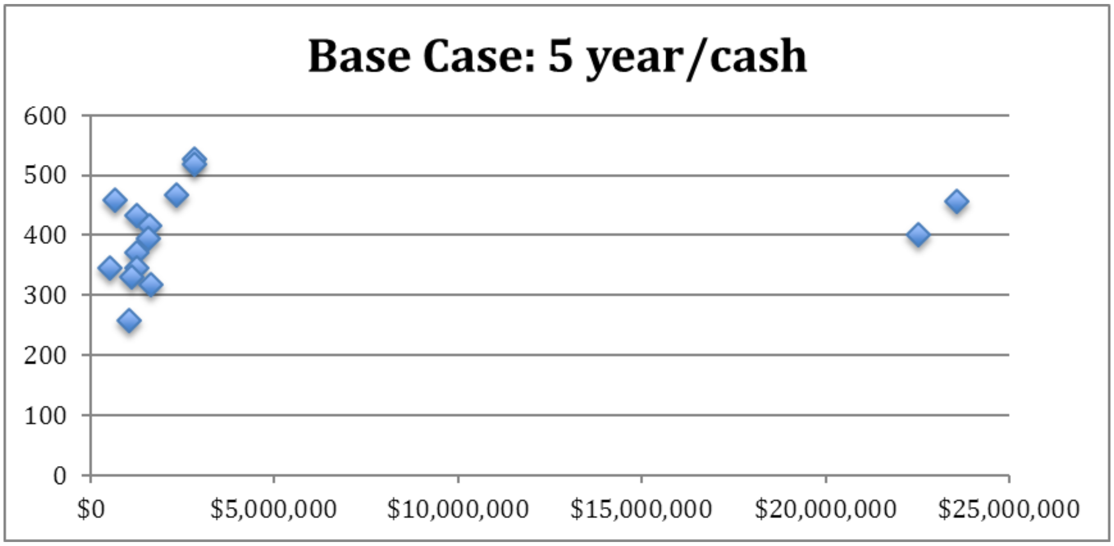

Career Promotion High: 5 year/cash

Package	Total Direct Cost	QALYs	Cost per QALY	
1	$2,874,131	558	$5,152	$60,810
2	$2,869,444	555	$5,171	Dom.
3	$2,347,845	484	$4,851	Dom.
4	$709,648	522	$1,359	$1,535
5	$23,594,809	472	$50,022	Dom.
6	$1,373,202	512	$2,680	Dom.
7	$1,604,154	426	$3,769	Dom.
8	$1,594,078	406	$3,927	Dom.
9	$22,596,002	493	$45,832	Dom.
10	$1,252,563	353	$3,550	Dom.
11	$606,611	455	$1,333	$1,333
12	$1,239,995	326	$3,803	Dom.
13	$1,114,205	337	$3,310	Dom.
14	$1,670,451	331	$5,054	Dom.
15	$1,047,792	286	$3,662	Dom.

Career Promotion Low: 5 year/cash

Package	Total Direct Cost	QALYs	Cost per QALY	
1	$2,759,392	485	$5,693	$518,118
2	$2,739,172	472	$5,805	Dom.
3	$2,347,845	484	$4,851	$12,755
4	$595,710	346	$1,724	$1,724
5	$23,594,809	472	$50,022	Dom.
6	$1,120,581	312	$3,594	Dom.
7	$1,604,154	426	$3,769	$12,604
8	$1,594,078	406	$3,927	Dom.
9	$22,453,929	251	$89,428	Dom.
10	$1,252,563	353	$3,550	Dom.
11	$426,541	149	$2,872	Dom.
12	$1,239,995	326	$3,803	Dom.
13	$1,114,205	337	$3,310	Dom.
14	$1,670,451	331	$5,054	Dom.
15	$1,047,792	286	$3,662	Dom.

Continuing Education (1) High: 5 year/cash

Package	Total Direct Cost	QALYs	Cost per QALY	
1	$2,867,016	553	$5,181	$23,739
2	$2,861,293	550	$5,205	Dom.
3	$2,395,281	524	$4,571	Dom.
4	$669,983	461	$1,454	$1,454
5	$23,617,799	517	$45,707	Dom.
6	$1,283,630	441	$2,909	Dom.
7	$1,636,266	489	$3,349	Dom.
8	$1,629,932	476	$3,423	Dom.
9	$22,543,898	404	$55,762	Dom.
10	$1,252,563	353	$3,550	Dom.
11	$536,504	336	$1,598	Dom.
12	$1,239,995	326	$3,803	Dom.
13	$1,114,205	337	$3,310	Dom.
14	$1,670,451	331	$5,054	Dom.
15	$1,077,302	396	$2,719	Dom.

Continuing Education (1) Low 5 year/cash

Package	Total Direct Cost	QALYs	Cost per QALY	
1	$2,785,045	501	$5,558	$52,464
2	$2,768,082	490	$5,646	Dom.
3	$2,270,175	418	$5,427	Dom.
4	$669,983	461	$1,454	$1,454
5	$23,557,651	399	$59,057	Dom.
6	$1,283,630	441	$2,909	Dom.
7	$1,554,706	329	$4,729	Dom.
8	$1,539,993	300	$5,134	Dom.
9	$22,543,898	404	$55,762	Dom.
10	$1,252,563	353	$3,550	Dom.
11	$536,504	336	$1,598	Dom.
12	$1,239,995	326	$3,803	Dom.
13	$1,114,205	337	$3,310	Dom.
14	$1,670,451	331	$5,054	Dom.
15	$1,008,441	139	$7,239	Dom.

Facility Quality High 5 year/cash

Package	Total Direct Cost	QALYs	Cost per QALY	
1	$2,836,963	534	$5,311	$29,523
2	$2,826,972	528	$5,356	Dom.
3	$2,347,845	484	$4,851	Dom.
4	$669,983	461	$1,454	$1,454
5	$23,623,361	528	$44,774	Dom.
6	$1,283,630	441	$2,909	Dom.
7	$1,604,154	426	$3,769	Dom.
8	$1,594,078	406	$3,927	Dom.
9	$22,595,904	493	$45,847	Dom.
10	$1,252,563	353	$3,550	Dom.
11	$536,504	336	$1,598	Dom.
12	$1,239,995	326	$3,803	Dom.
13	$1,114,205	337	$3,310	Dom.
14	$1,670,451	331	$5,054	Dom.
15	$1,047,792	286	$3,662	Dom.

Quality Low 5 year/cash

Package	Total Direct Cost	QALYs	Cost per QALY	
1	$2,836,963	534	$5,311	$29,523
2	$2,826,972	528	$5,356	Dom.
3	$2,347,845	484	$4,851	Dom.
4	$669,983	461	$1,454	$1,454
5	$23,540,576	365	$64,416	Dom.
6	$1,283,630	441	$2,909	Dom.
7	$1,604,154	426	$3,769	Dom.
8	$1,594,078	406	$3,927	Dom.
9	$22,454,220	252	$89,253	Dom.
10	$1,252,563	353	$3,550	Dom.
11	$536,504	336	$1,598	Dom.
12	$1,239,995	326	$3,803	Dom.
13	$1,114,205	337	$3,310	Dom.
14	$1,670,451	331	$5,054	Dom.
15	$1,047,792	286	$3,662	Dom.

Transportation Both High 5 year/cash

Package	Total Direct Cost	QALYs	Cost per QALY	
1	$2,857,495	547	$5,222	$40,813
2	$2,826,972	528	$5,356	Dom.
3	$2,347,845	484	$4,851	Dom.
4	$691,537	494	$1,399	$1,399
5	$23,610,391	502	$47,013	Dom.
6	$1,283,630	441	$2,909	Dom.
7	$1,625,787	468	$3,474	Dom.
8	$1,594,078	406	$3,927	Dom.
9	$22,543,898	404	$55,762	Dom.
10	$1,280,361	412	$3,107	Dom.
11	$536,504	336	$1,598	Dom.
12	$1,239,995	326	$3,803	Dom.
13	$1,114,205	337	$3,310	Dom.
14	$1,670,451	331	$5,054	Dom.
15	$1,047,792	286	$3,662	Dom.

Transportation Both Low 5 year/cash

Package	Total Direct Cost	QALYs	Cost per QALY	
1	$2,808,137	516	$5,444	$21,720
2	$2,826,972	528	$5,356	Dom.
3	$2,347,845	484	$4,851	Dom.
4	$641,123	416	$1,541	$1,541
5	$23,573,797	431	$54,756	Dom.
6	$1,283,630	441	$2,909	Dom.
7	$1,575,832	370	$4,257	Dom.
8	$1,594,078	406	$3,927	Dom.
9	$22,543,898	404	$55,762	Dom.
10	$1,217,824	279	$4,369	Dom.
11	$536,504	336	$1,598	Dom.
12	$1,239,995	326	$3,803	Dom.
13	$1,114,205	337	$3,310	Dom.
14	$1,670,451	331	$5,054	Dom.
15	$1,047,792	286	$3,662	Dom.

50% increase in salary (all packages vs. 0% increase in minimum package): 5 year/cash
[Applies to **new** rural physicians: Post 2012 entrants only]

Package	Total Direct Cost	QALYs	Cost per QALY	ICER
1	$3,092,061	565	$5,475	$91,468
2	$3,088,615	563	$5,488	Dom.
3	$2,628,745	549	$4,790	Dom.
4	$924,573	541	$1,709	$2,171
5	$23,835,557	545	$43,756	Dom.
6	$1,602,535	534	$2,999	Dom.
7	$1,857,449	529	$3,512	Dom.
8	$1,852,539	522	$3,550	Dom.
9	$22,811,750	521	$43,767	Dom.
10	$1,518,023	502	$3,026	Dom.
11	$824,416	495	$1,666	$1,666
12	$1,511,051	491	$3,077	Dom.
13	$1,360,398	495	$2,747	Dom.
14	$2,017,130	493	$4,093	Dom.
15	$1,288,884	474	$2,718	Dom.

50% increase in salary (all packages vs. 0% increase in minimum package): 5 year/cash
[Applies to **all** rural physicians]

Package	Total Direct Cost	QALYs	Cost per QALY	ICER
1	$3,842,725	565	$6,804	$91,468
2	$3,839,279	563	$6,822	Dom.
3	$3,379,409	549	$6,158	Dom.
4	$1,675,236	541	$3,096	$3,096
5	$24,586,221	545	$45,134	Dom.
6	$2,353,198	534	$4,404	Dom.
7	$2,608,113	529	$4,931	Dom.
8	$2,603,203	522	$4,989	Dom.
9	$23,562,413	521	$45,207	Dom.
10	$2,268,687	502	$4,522	Dom.
11	$1,575,080	495	$3,182	Dom.
12	$2,261,715	491	$4,606	Dom.
13	$2,111,062	495	$4,262	Dom.
14	$2,767,794	493	$5,616	Dom.
15	$2,039,548	474	$4,301	Dom.

Rural Share of MDs is 50% larger: 5 year/cash

Package	Total Direct Cost	QALYs	Cost per QALY	ICER
1	$4,255,445	751	$5,665	$31,491
2	$4,240,457	742	$5,713	Dom.
3	$3,521,768	681	$5,175	Dom.
4	$1,004,975	648	$1,551	$1,551
5	$24,408,638	663	$36,798	Dom.
6	$1,925,446	620	$3,103	Dom.
7	$2,406,231	599	$4,020	Dom.
8	$2,391,116	571	$4,189	Dom.
9	$22,832,272	569	$40,159	Dom.
10	$1,878,845	496	$3,786	Dom.
11	$804,757	472	$1,704	Dom.
12	$1,859,993	458	$4,057	Dom.
13	$1,671,307	473	$3,531	Dom.
14	$2,505,677	465	$5,391	Dom.
15	$1,571,687	402	$3,906	Dom.

Rural Share of MDs is 50% smaller: 5 year/cash

Package	Total Direct Cost	QALYs	Cost per QALY	ICER
1	$1,418,482	285	$4,982	$27,696
2	$1,413,486	281	$5,024	Dom.
3	$1,173,923	258	$4,551	Dom.
4	$334,992	246	$1,364	$1,364
5	$22,780,980	251	$90,613	Dom.
6	$641,815	235	$2,729	Dom.
7	$802,077	227	$3,535	Dom.
8	$797,039	216	$3,684	Dom.
9	$22,255,524	215	$103,280	Dom.
10	$626,282	188	$3,330	Dom.
11	$268,252	179	$1,499	Dom.
12	$619,998	174	$3,568	Dom.
13	$557,102	179	$3,105	Dom.
14	$835,226	176	$4,741	Dom.
15	$523,896	153	$3,435	Dom.

Discount Rates 5% (Health and Finance, Base Case 3%)
5 year/cash

Package	Total Direct Cost	QALYs	Cost per QALY	ICER
1	$2,707,125	359	$7,537	$41,930
2	$2,697,585	355	$7,601	Dom.
3	$2,240,574	325	$6,886	Dom.
4	$637,798	310	$2,059	$2,059
5	$22,677,459	317	$71,503	Dom.
6	$1,228,218	297	$4,140	Dom.
7	$1,526,389	286	$5,333	Dom.
8	$1,516,843	273	$5,558	Dom.
9	$21,677,990	272	$79,746	Dom.
10	$1,182,743	237	$4,985	Dom.
11	$510,868	226	$2,263	Dom.
12	$1,170,873	219	$5,341	Dom.
13	$1,051,428	226	$4,645	Dom.
14	$1,586,545	222	$7,139	Dom.
15	$988,494	192	$5,138	Dom.

Attrition Rate 5.0% (Base Case 2.5%)
5 year/cash

Package	Total Direct Cost	QALYs	Cost per QALY	ICER
1	$2,551,550	348	$7,327	$40,461
2	$2,542,390	344	$7,389	Dom.
3	$2,102,580	315	$6,664	Dom.
4	$615,465	300	$2,049	$2,049
5	$22,571,509	308	$73,402	Dom.
6	$1,177,509	288	$4,094	Dom.
7	$1,421,173	277	$5,122	Dom.
8	$1,411,941	265	$5,336	Dom.
9	$21,658,577	264	$82,174	Dom.
10	$1,092,843	230	$4,751	Dom.
11	$492,810	219	$2,251	Dom.
12	$1,081,387	213	$5,088	Dom.
13	$965,704	219	$4,401	Dom.
14	$1,475,221	215	$6,846	Dom.
15	$904,833	187	$4,850	Dom.

Attrition Rate 1.0% (Base Case 2.5%)
5 year/cash

Package	Total Direct Cost	QALYs	Cost per QALY	ICER
1	$2,805,078	366	$7,667	$42,835
2	$2,795,303	362	$7,732	Dom.
3	$2,327,637	331	$7,022	Dom.
4	$651,614	316	$2,065	$2,065
5	$22,744,525	323	$70,401	Dom.
6	$1,259,758	302	$4,169	Dom.
7	$1,593,002	292	$5,464	Dom.
8	$1,583,261	278	$5,695	Dom.
9	$21,690,001	277	$78,328	Dom.
10	$1,239,974	242	$5,131	Dom.
11	$522,041	230	$2,270	Dom.
12	$1,227,848	223	$5,498	Dom.
13	$1,106,077	231	$4,797	Dom.
14	$1,657,183	226	$7,320	Dom.
15	$1,041,869	196	$5,316	Dom.

Attrition Rate 5.0% (Base Case 2.5%)
30 year/cash

Package	Total Direct Cost	QALYs	Cost per QALY	ICER
1	$27,087,601	3,856	$7,026	$31,979
2	$26,952,566	3,810	$7,075	Dom.
3	$22,995,132	3,493	$6,583	Dom.
4	$10,146,015	3,326	$3,051	$3,051
5	$58,539,749	3,405	$17,195	Dom.
6	$12,651,031	3,185	$3,973	Dom.
7	$17,717,800	3,072	$5,767	Dom.
8	$17,438,201	2,930	$5,952	Dom.
9	$48,244,835	2,918	$16,533	Dom.
10	$16,392,459	2,547	$6,436	Dom.
11	$7,511,641	2,423	$3,100	Dom.
12	$16,013,788	2,353	$6,805	Dom.
13	$12,666,265	2,430	$5,213	Dom.
14	$15,006,629	2,386	$6,290	Dom.
15	$10,809,961	2,065	$5,234	Dom.

Attrition Rate 1.0% (Base Case 2.5%)
30 year/cash

Package	Total Direct Cost	QALYs	Cost per QALY	ICER
1	$38,171,913	4,923	$7,753	$36,051
2	$37,987,653	4,865	$7,809	Dom.
3	$32,405,262	4,460	$7,265	Dom.
4	$13,784,124	4,247	$3,246	$3,246
5	$65,958,833	4,347	$15,172	Dom.
6	$17,554,054	4,067	$4,317	Dom.
7	$24,920,506	3,923	$6,352	Dom.
8	$24,548,147	3,741	$6,562	Dom.
9	$51,181,806	3,726	$13,736	Dom.
10	$23,230,278	3,252	$7,143	Dom.
11	$10,243,679	3,095	$3,310	Dom.
12	$22,725,642	3,005	$7,563	Dom.
13	$18,269,301	3,102	$5,889	Dom.
14	$21,777,306	3,046	$7,149	Dom.
15	$15,797,045	2,637	$5,990	Dom.

Public Sector Share 85% (Base Case 70%)
5 Year/Cash

Package	Total Direct Cost	QALYs	Cost per QALY	ICER
1	$3,302,692	426	$7,754	$43,135
2	$3,291,054	421	$7,820	Dom.
3	$2,733,500	386	$7,084	Dom.
4	$778,113	367	$2,118	$2,118
5	$23,018,164	376	$61,200	Dom.
6	$1,498,426	352	$4,259	Dom.
7	$1,862,195	339	$5,487	Dom.
8	$1,850,549	324	$5,718	Dom.
9	$21,798,811	322	$67,619	Dom.
10	$1,442,946	281	$5,129	Dom.
11	$623,259	268	$2,328	Dom.
12	$1,428,465	260	$5,495	Dom.
13	$1,282,742	268	$4,779	Dom.
14	$1,935,585	264	$7,344	Dom.
15	$1,205,963	228	$5,285	Dom.

Public Sector Share 55% (Base Case 70%)
5 Year/Cash

Package	Total Direct Cost	QALYs	Cost per QALY	ICER
1	$2,138,628	291	$7,337	$40,816
2	$2,131,092	288	$7,399	Dom.
3	$1,770,053	264	$6,703	Dom.
4	$503,860	251	$2,004	$2,004
5	$22,352,241	257	$86,842	Dom.
6	$970,292	241	$4,030	Dom.
7	$1,205,848	232	$5,192	Dom.
8	$1,198,306	221	$5,410	Dom.
9	$21,562,660	221	$97,739	Dom.
10	$934,367	193	$4,853	Dom.
11	$403,586	183	$2,203	Dom.
12	$924,990	178	$5,199	Dom.
13	$830,628	184	$4,522	Dom.
14	$1,253,371	180	$6,949	Dom.
15	$780,911	156	$5,001	Dom.

Exhibit 5: Student Sub-Analyses

Mixed Logit: Full Student Sample (Base Case)

```
Mixed logit model                                  Number of obs   =
7896
                                                   LR chi2(9)      =
154.97
Log likelihood = -2139.5089                        Prob > chi2     =
0.0000

----------------------------------------------------------------------
---------
      choice |      Coef.   Std. Err.      z    P>|z|     [95% Conf.
Interval]
-------------+--------------------------------------------------------
---------
Mean         |
    salary_a |   2.464348   .1651824    14.92   0.000     2.140597
2.7881
 house_allow |   .7128856   .0653482    10.91
0.000     .5848055    .8409658
  house_prov |   .6812241   .0644884    10.56
0.000     .5548291    .8076191
 career_pro0 |   .6697503    .075209     8.91
0.000     .5223434    .8171572
 career_pro1 |   .4643231   .0609779     7.61
0.000     .3448086    .5838376
     coned_1 |   1.085816   .0757079    14.34   0.000     .9374313
1.234201
     coned_2 |    .619682   .0619217    10.01
0.000     .4983177    .7410463
   quality_a |   .4051129   .0615178     6.59
0.000     .2845403    .5256856
   trans_off |   .6583239    .063668    10.34
0.000      .533537    .7831109
  trans_both |   .8001244   .0672841    11.89
0.000     .6682499    .9319989
-------------+--------------------------------------------------------
---------
SD           |
 house_allow |  -.2387377   .1299566    -1.84   0.066    -
.4934479    .0159725
  house_prov |   .2281767   .1768794     1.29   0.197    -
.1185006     .574854
 career_pro0 |    .802309   .0843734     9.51
0.000     .6369403    .9676778
 career_pro1 |  -.0966943   .1132604    -0.85   0.393    -
.3186806    .1252921
     coned_1 |   .5939945     .09756     6.09
0.000     .4027804    .7852086
     coned_2 |   .1345518   .1304097     1.03   0.302    -
.1210464    .3901501
   quality_a |   .8001923   .0683078    11.71
0.000     .6663115    .9340731
   trans_off |   .1216367   .1055813     1.15   0.249    -
.0852988    .3285722
  trans_both |   .3676548   .1082969     3.39
0.001     .1553968    .5799127
```

Mixed Logit: Exclude 5 post graduates

```
Mixed logit model                                  Number of obs   =
7776
                                                   LR chi2(9)      =
136.65
Log likelihood = -2112.4691                        Prob > chi2     =
0.0000

------------------------------------------------------------------------
---------
      choice |      Coef.   Std. Err.      z    P>|z|     [95% Conf.
Interval]
-------------+----------------------------------------------------------
---------
Mean         |
    salary_a |    2.40321   .1635332    14.70   0.000     2.082691
2.723729
 house_allow |   .7030183   .0658632    10.67
0.000     .5739288    .8321078
  house_prov |   .6685253   .0634792    10.53
0.000     .5441084    .7929423
 career_pro0 |   .6710994   .0751486     8.93
0.000     .5238109    .8183879
 career_pro1 |   .4534158   .0603394     7.51
0.000     .3351528    .5716787
     coned_1 |   1.076748   .0740644    14.54   0.000     .9315844
1.221911
     coned_2 |   .6299451   .0632377     9.96
0.000     .5060016    .7538886
   quality_a |   .4253028   .0610296     6.97
0.000     .3056869    .5449187
   trans_off |   .6351125   .0629149    10.09
0.000     .5118015    .7584236
  trans_both |    .777382   .0659015    11.80
0.000     .6482175    .9065465
-------------+----------------------------------------------------------
---------
SD           |
 house_allow |  -.2938655   .1270895    -2.31   0.021    -.5429564
-.0447746
  house_prov |  -.1497428   .2066864    -0.72   0.469    -
.5548408    .2553552
 career_pro0 |   .7692723   .0864556     8.90
0.000     .5998224    .9387222
 career_pro1 |   -.010899   .1216263    -0.09   0.929    -
.2492821    .2274842
     coned_1 |   .5710689   .0993049     5.75
0.000     .3764348     .765703
     coned_2 |   .2154317   .1214111     1.77   0.076    -
.0225298    .4533932
   quality_a |   .7614972   .0693759    10.98
0.000     .6255229    .8974714
   trans_off |  -.0112159   .1392641    -0.08   0.936    -
.2841685    .2617366
  trans_both |  -.3091547   .1364411    -2.27   0.023    -.5765743
-.041735
```

Mixed Logit: Students with a rural rotation (80% of sample)

Mixed logit model | Number of obs = 6312
LR chi2(9) = 136.10
Log likelihood = -1682.3466 | Prob > chi2 = 0.0000

choice	Coef.	Std. Err.	z	P>|z|	[95% Conf.	Interval]
**Mean**						
salary_a	2.647307	.1918799	13.80	0.000	2.27123	3.023385
house_allow	.7684185	.0747486	10.28	0.000	.621914	.9149231
house_prov	.7091355	.0749866	9.46	0.000	.5621645	.8561065
career_pro0	.7054351	.0853102	8.27	0.000	.5382302	.87264
career_pro1	.4747675	.06907	6.87	0.000	.3393928	.6101421
coned_1	1.128331	.0869693	12.97	0.000	.9578747	1.298788
coned_2	.6483637	.0706492	9.18	0.000	.5098939	.7868335
quality_a	.4137105	.0751099	5.51	0.000	.2664978	.5609231
trans_off	.7197886	.0734803	9.80	0.000	.5757698	.8638073
trans_both	.8757307	.0788255	11.11	0.000	.7212355	1.030226
**SD**						
house_allow	.1928368	.1345272	1.43	0.152	-.0708317	.4565054
house_prov	.317262	.1225525	2.59	0.010	.0770636	.5574604
career_pro0	.7777561	.1055455	7.37	0.000	.5708906	.9846216
career_pro1	.038939	.1185751	0.33	0.743	-.193464	.271342
coned_1	.6581523	.1134744	5.80	0.000	.4357466	.8805579
coned_2	.101741	.1846469	0.55	0.582	-.2601603	.4636423
quality_a	.9040246	.0834969	10.83	0.000	.7403738	1.067676
trans_off	.1369439	.1143515	1.20	0.231	-.0871809	.3610687
trans_both	-.3935987	.130768	-3.01	0.003	-.6498992	-.1372982

Mixed Logit: Students without a rural rotation (20% of sample)

```
Mixed logit model                                 Number of obs   =
1584
                                                  LR chi2(9)      =
19.89
Log likelihood = -451.89622                       Prob > chi2     =
0.0186

----------------------------------------------------------------------
---------
      choice |      Coef.   Std. Err.      z    P>|z|     [95% Conf.
Interval]
-------------+--------------------------------------------------------
---------
Mean         |
    salary_a |   1.982279   .3416212     5.80   0.000     1.312714
2.651844
 house_allow |   .5474585   .1531754     3.57
0.000     .2472402    .8476768
  house_prov |   .6293368   .1435931     4.38
0.000     .3478995    .9107741
 career_pro0 |   .6360255   .1735989     3.66
0.000      .295778    .9762731
 career_pro1 |   .4413633   .1394343     3.17
0.002     .1680771    .7146496
     coned_1 |    .968768   .1575231     6.15   0.000     .6600284
1.277508
     coned_2 |   .6025683   .1479955     4.07
0.000     .3125025    .8926342
   quality_a |   .5243925   .1178151     4.45
0.000      .293479    .7553059
   trans_off |   .4023324   .1379166     2.92
0.004     .1320209    .6726439
  trans_both |   .5608151   .1392885     4.03
0.000     .2878147    .8338155
-------------+--------------------------------------------------------
---------
SD           |
 house_allow |   .5851308   .2018341     2.90
0.004     .1895433    .9807184
  house_prov |  -.3623426   .2578505    -1.41   0.160    -
.8677204    .1430352
 career_pro0 |   .8646406   .1842186     4.69   0.000     .5035788
1.225702
 career_pro1 |    .326574   .2347789     1.39   0.164    -
.1335843    .7867322
     coned_1 |  -.4753678   .1970898    -2.41   0.016    -.8616566
-.0890789
     coned_2 |     .38697   .2183261     1.77   0.076    -
.0409412    .8148813
   quality_a |   .5301237   .1516232     3.50
0.000     .2329478    .8272997
   trans_off |   -.248663   .2171374    -1.15   0.252    -
.6742446    .1769185
  trans_both |  -.2202131   .2827236    -0.78   0.436    -
.7743411     .333915
```

Mixed Logit: 5th Year Students (60% of sample)

Mixed logit model — Number of obs = 4728

LR chi2(9) = 122.69

Log likelihood = -1260.4612 — Prob > chi2 = 0.0000

choice	Coef.	Std. Err.	z	P>|z|	[95% Conf.	Interval]
**Mean**						
salary_a	2.434083	.213492	11.40	0.000	2.015646	2.852519
house_allow	.7204169	.0843649	8.54	0.000	.5550648	.885769
house_prov	.7367918	.0874355	8.43	0.000	.5654214	.9081622
career_pro0	.7476193	.102741	7.28	0.000	.5462507	.9489879
career_pro1	.53733	.0826159	6.50	0.000	.3754058	.6992542
coned_1	1.132774	.0984656	11.50	0.000	.9397855	1.325763
coned_2	.6164733	.08124	7.59	0.000	.4572458	.7757008
quality_a	.4814716	.0859484	5.60	0.000	.3130157	.6499275
trans_off	.6112589	.0829096	7.37	0.000	.448759	.7737587
trans_both	.8739236	.0879655	9.93	0.000	.7015144	1.046333
**SD**						
house_allow	.0760743	.2774146	0.27	0.784	-.4676482	.6197969
house_prov	-.3285843	.1413583	-2.32	0.020	-.6056415	-.0515271
career_pro0	.9147909	.1146049	7.98	0.000	.6901695	1.139412
career_pro1	-.2915786	.1310219	-2.23	0.026	-.5483767	-.0347804
coned_1	.5552177	.1215066	4.57	0.000	.3170691	.7933662
coned_2	-.103446	.1894803	-0.55	0.585	-.4748205	.2679286
quality_a	.9286182	.0973103	9.54	0.000	.7378936	1.119343
trans_off	.0712601	.1350448	0.53	0.598	-.1934229	.3359431
trans_both	.195609	.164628	1.19	0.235	-.1270559	.5182739

Mixed Logit: 6th Year Students (39% of sample)

```
Mixed logit model                                 Number of obs   =
3048
                                                  LR chi2(9)      =
37.55
Log likelihood = -835.86547                       Prob > chi2     =
0.0000

----------------------------------------------------------------------
---------
      choice |      Coef.   Std. Err.      z    P>|z|     [95% Conf.
Interval]
-------------+--------------------------------------------------------
---------
Mean         |
    salary_a |   2.583317   .2673582     9.66   0.000     2.059305
3.10733
 house_allow |   .6970369    .104441     6.67
0.000     .4923364    .9017375
  house_prov |   .6083566   .1058981     5.74
0.000     .4008001    .8159131
 career_pro0 |   .5555686   .1110937     5.00
0.000      .337829    .7733082
 career_pro1 |   .3685549   .0964613     3.82
0.000     .1794943    .5576155
     coned_1 |   1.088713   .1284501     8.48   0.000     .8369558
1.340471
     coned_2 |   .6683111   .0992012     6.74
0.000     .4738803    .8627419
   quality_a |   .3276665   .0854824     3.83
0.000      .160124    .4952089
   trans_off |   .7039727   .1039184     6.77
0.000     .5002965    .9076489
  trans_both |   .7275349   .1117047     6.51
0.000     .5085978    .9464721
-------------+--------------------------------------------------------
---------
SD           |
 house_allow |  -.2442628   .2185407    -1.12   0.264    -
.6725947    .1840691
  house_prov |   .4160804   .1477197     2.82
0.005     .1265551    .7056057
 career_pro0 |   .6507417   .1368002     4.76
0.000     .3826183    .9188652
 career_pro1 |  -.0365204   .1802166    -0.20   0.839    -
.3897385    .3166977
     coned_1 |   .7759431   .1545473     5.02   0.000      .4730359
1.07885
     coned_2 |   .1834006   .2599449     0.71   0.480    -
.3260821    .6928833
   quality_a |   .5530906   .1102848     5.02
0.000     .3369363    .7692449
   trans_off |   .2045537   .1729107     1.18   0.237    -
.1343451    .5434525
  trans_both |   .5079185   .1730487     2.94
0.003     .1687493    .8470877
```

Exhibit 6: Students vs. Currently Practicing Physicians

Mixed Logit: Full Student Sample (Base Case)

```
Mixed logit model                                   Number of obs   =
7896
                                                    LR chi2(9)      =
154.97
Log likelihood = -2139.5089                         Prob > chi2     =
0.0000

----------------------------------------------------------------------
---------
      choice |      Coef.   Std. Err.      z    P>|z|     [95% Conf.
Interval]
-------------+--------------------------------------------------------
---------
Mean         |
    salary_a |   2.464348   .1651824    14.92   0.000     2.140597
2.7881
 house_allow |   .7128856   .0653482    10.91
0.000     .5848055    .8409658
  house_prov |   .6812241   .0644884    10.56
0.000     .5548291    .8076191
 career_pro0 |   .6697503    .075209     8.91
0.000     .5223434    .8171572
 career_pro1 |   .4643231   .0609779     7.61
0.000     .3448086    .5838376
     coned_1 |   1.085816   .0757079    14.34   0.000     .9374313
1.234201
     coned_2 |    .619682   .0619217    10.01
0.000     .4983177    .7410463
   quality_a |   .4051129   .0615178     6.59
0.000     .2845403    .5256856
   trans_off |   .6583239    .063668    10.34
0.000      .533537    .7831109
  trans_both |   .8001244   .0672841    11.89
0.000     .6682499    .9319989
-------------+--------------------------------------------------------
---------
SD           |
 house_allow |  -.2387377   .1299566    -1.84   0.066    -
.4934479    .0159725
  house_prov |   .2281767   .1768794     1.29   0.197    -
.1185006     .574854
 career_pro0 |    .802309   .0843734     9.51
0.000     .6369403    .9676778
 career_pro1 |  -.0966943   .1132604    -0.85   0.393    -
.3186806    .1252921
     coned_1 |   .5939945     .09756     6.09
0.000     .4027804    .7852086
     coned_2 |   .1345518   .1304097     1.03   0.302    -
.1210464    .3901501
   quality_a |   .8001923   .0683078    11.71
0.000     .6663115    .9340731
   trans_off |   .1216367   .1055813     1.15   0.249    -
.0852988    .3285722
  trans_both |   .3676548   .1082969     3.39
0.001     .1553968    .5799127
```

Mixed Logit for Currently Practicing Physicians

Mixed logit model Number of obs = 2490

LR chi2(9) = 32.03

Log likelihood = -695.27021 Prob > chi2 = 0.0002

choice	Coef.	Std. Err.	z	P>|z|	[95% Conf.	Interval]
**Mean**						
salary_a	1.797695	.2635513	6.82	0.000	1.281144	2.314246
house_allow	.561389	.1052834	5.33	0.000	.3550373	.7677407
house_prov	.6712536	.1196097	5.61	0.000	.4368229	.9056842
career_pro0	.7488748	.1193367	6.28	0.000	.5149792	.9827704
career_pro1	.45193	.1045934	4.32	0.000	.2469307	.6569293
coned_1	.5764921	.1167708	4.94	0.000	.3476255	.8053588
coned_2	.2919869	.1019945	2.86	0.004	.0920814	.4918924
quality_a	.6284875	.1040989	6.04	0.000	.4244573	.8325176
trans_off	.7896519	.1100237	7.18	0.000	.5740095	1.005294
trans_both	.7967181	.1112176	7.16	0.000	.5787355	1.014701
**SD**						
house_allow	-.0355474	.2599915	-0.14	0.891	-.5451214	.4740266
house_prov	.5212482	.1432721	3.64	0.000	.24044	.8020563
career_pro0	.4891141	.166672	2.93	0.003	.1624429	.8157852
career_pro1	.0889262	.2110618	0.42	0.674	-.3247473	.5025997
coned_1	.439448	.1838397	2.39	0.017	.0791287	.7997672
coned_2	.0241983	.2256422	0.11	0.915	-.4180523	.4664488
quality_a	.7039749	.1234537	5.70	0.000	.4620101	.9459398
trans_off	.1798321	.1558141	1.15	0.248	-.125558	.4852221
trans_both	-.2145643	.2034527	-1.05	0.292	-.6133242	.1841956

Attribute	Relative Coefficients (vs. Salary) MDs	Students	Relative Comparison (vs. Students)
Salary	1.00	1.00	--
Housing (Allowance)	0.31	0.29	Similar
Housing (Provision)	0.37	0.28	Larger
Career Promotion (Immediate)	0.42	0.27	Larger
Career Promotion (1 Year)	0.25	0.19	Larger
Continuing Education (1 Year)	0.32	0.44	Smaller
Continuing Education (2 Year)	0.16	0.25	Smaller
Facility Quality	0.35	0.16	Larger
Transportation (Official Use Only)	0.44	0.27	Larger
Transportation (All)	0.44	0.32	Larger

References

1. Ryan M, Gerard K, Amaya-Amaya M. Using Discrete Choice Experiments to Value Health and Health Care. Netherlands: Springer Science & Business Media; 2007.
2. Vujicic M, Alfano M, Shengalia B, Witter S. Attracting Doctors and Medical Students to Rural Vietnam: Insights from a Discrete Choice Experiment. World Bank Health and Nutrition Policy (HNP) Discussion Paper. Washington DC; 2010.
3. Jaskiewicz W, Deussom R, Wurts L, Mgomella G. Rapid Retention Survey Toolkit: Designing Evidence-Based Incentives for Health Workers. Washington, DC: CapacityPlus; 2012.
4. Ryan M, Kolstad J, Rockers PC, Dolea C. *How to Conduct a Discrete Choice Experiment for Health Workforce Recruitment and Retention in Remote and Rural Area: A User's Guide for Policy Makers and Researchers.* Geneva: World Health Organization; 2011.
5. Claxton K, Soares M, Rice N, et al. Methods for the Estimation of the NICE Cost Effectiveness Threshold. York, UK: University of York, Centre for Health Economics; 2013.
6. Fryback D JH. *Do large national surveys yield equivalent population norms for health related quality of life measures?* San Francisco, CA: Society for Medical Decision Making; 2005.
7. Hoi L, Chuc N, Lindholm L. Health-related quality of life, and its determinants, among older people in rural Vietnam. *BMC Public Health*. 2010;10:549.
8. Hoi L, Phuc H, Dung T, Chuc N, Lindholm L. Remaining life expectancy among older people in a rural area of Vietnam: trends and socioeconomic inequalities during a period of multiple transitions. *BMC Public Health.* 2009;9:471.
